# Six Novel Susceptibility Loci for Early-Onset Androgenetic Alopecia and Their Unexpected Association with Common Diseases

**DOI:** 10.1371/journal.pgen.1002746

**Published:** 2012-05-31

**Authors:** Rui Li, Felix F. Brockschmidt, Amy K. Kiefer, Hreinn Stefansson, Dale R. Nyholt, Kijoung Song, Sita H. Vermeulen, Stavroula Kanoni, Daniel Glass, Sarah E. Medland, Maria Dimitriou, Dawn Waterworth, Joyce Y. Tung, Frank Geller, Stefanie Heilmann, Axel M. Hillmer, Veronique Bataille, Sibylle Eigelshoven, Sandra Hanneken, Susanne Moebus, Christine Herold, Martin den Heijer, Grant W. Montgomery, Panos Deloukas, Nicholas Eriksson, Andrew C. Heath, Tim Becker, Patrick Sulem, Massimo Mangino, Peter Vollenweider, Tim D. Spector, George Dedoussis, Nicholas G. Martin, Lambertus A. Kiemeney, Vincent Mooser, Kari Stefansson, David A. Hinds, Markus M. Nöthen, J. Brent Richards

**Affiliations:** 1 Departments of Medicine, Human Genetics, Epidemiology, and Biostatistics, Lady Davis Institute, Jewish General Hospital, McGill University, Montreal, Quebec, Canada; 2 Institute of Human Genetics, University of Bonn, Bonn, Germany; 3 Department of Genomics, Life and Brain Center, University of Bonn, Bonn, Germany; 4 23andMe, Mountain View, California, United States of America; 5 deCODE genetics, Reykjavík, Iceland; 6 Queensland Institute of Medical Research, Brisbane, Australia; 7 Genetics Division, GlaxoSmithKline, King of Prussia, Pennsylvania, United States of America; 8 Department of Epidemiology, Biostatistics, and HTA, Radboud University Nijmegen Medical Centre, Nijmegen, The Netherlands; 9 Department of Genetics, Radboud University Nijmegen Medical Centre, Nijmegen, The Netherlands; 10 Genetics of Complex Traits in Humans, Wellcome Trust Sanger Institute, Wellcome Trust Genome Campus, Hinxton, United Kingdom; 11 Twin Research and Genetic Epidemiology, King's College London, London, United Kingdom; 12 Department of Dietetics-Nutrition, Harokopio University, Athens, Greece; 13 Department of Epidemiology Research, Statens Serum Institute, Copenhagen, Denmark; 14 Genome Technology and Biology, Genome Institute of Singapore, Singapore, Singapore; 15 Department of Dermatology, University of Düsseldorf, Düsseldorf, Germany; 16 Institute for Medical Informatics, Biometry, and Epidemiology, University Clinic Essen, University Duisburg-Essen, Essen, Germany; 17 German Center for Neurodegenerative Diseases (DZNE), Bonn, Germany; 18 Department of Endocrinology, Radboud University Nijmegen Medical Centre, Nijmegen, The Netherlands; 19 Washington University Medical School, St. Louis, Missouri, United States of America; 20 Institute for Medical Biometry, Informatics, and Epidemiology, University of Bonn, Bonn, Germany; 21 Department of Internal Medicine, CHUV University Hospital, Lausanne, Switzerland; 22 Department of Urology, Radboud University Nijmegen Medical Centre, Nijmegen, The Netherlands; 23 Comprehensive Cancer Centre of the Netherlands (IKNL), Nijmegen, The Netherlands; Vanderbilt University, United States of America

## Abstract

Androgenetic alopecia (AGA) is a highly heritable condition and the most common form of hair loss in humans. Susceptibility loci have been described on the X chromosome and chromosome 20, but these loci explain a minority of its heritable variance. We conducted a large-scale meta-analysis of seven genome-wide association studies for early-onset AGA in 12,806 individuals of European ancestry. While replicating the two AGA loci on the X chromosome and chromosome 20, six novel susceptibility loci reached genome-wide significance (p = 2.62×10^−9^–1.01×10^−12^). Unexpectedly, we identified a risk allele at 17q21.31 that was recently associated with Parkinson's disease (PD) at a genome-wide significant level. We then tested the association between early-onset AGA and the risk of PD in a cross-sectional analysis of 568 PD cases and 7,664 controls. Early-onset AGA cases had significantly increased odds of subsequent PD (OR = 1.28, 95% confidence interval: 1.06–1.55, p = 8.9×10^−3^). Further, the AGA susceptibility alleles at the 17q21.31 locus are on the H1 haplotype, which is under negative selection in Europeans and has been linked to decreased fertility. Combining the risk alleles of six novel and two established susceptibility loci, we created a genotype risk score and tested its association with AGA in an additional sample. Individuals in the highest risk quartile of a genotype score had an approximately six-fold increased risk of early-onset AGA [odds ratio (OR) = 5.78, p = 1.4×10^−88^]. Our results highlight unexpected associations between early-onset AGA, Parkinson's disease, and decreased fertility, providing important insights into the pathophysiology of these conditions.

## Author Summary

While most genome-wide association studies (GWAS) focus on the identification of susceptibility loci for a specific disease, this hypothesis-free approach also enables the identification of unexpected associations between different diseases by taking advantage of the previously published GWAS associations. Androgenetic Alopecia (AGA, also known as male pattern baldness) is the most common type of hair loss in humans. Parkinson's disease is reported to occur more commonly in men than in women; however, there are no studies investigating the link between AGA and Parkinson's disease. Here, we show that a specific genetic locus, chromosome 17q21.31, which is associated with Parkinson's disease, is also a susceptibility locus for early-onset AGA. We further investigate the association between early-onset AGA and Parkinson's disease, irrespective of genotype, directly in a large-scale web-based study. We find that men with early-onset AGA have 28% higher risk of developing Parkinson's disease. The early-onset AGA locus on chromosome 17q21.31 has also been linked to decreased fertility previously. Future studies of this locus may implicate novel biological pathways affecting these three conditions.

## Introduction

A main advantage of genome-wide association (GWA) studies is that their hypothesis-free scan of the genome for genes associated with common disease enables identification of novel associations between different diseases and pathways. For example, a pioneering GWA study demonstrated that susceptibility alleles for age-related macular degeneration were found in the complement pathway [1], giving rise to novel insights into the etiology and treatment of this condition [2].

Through previous GWA studies, we have identified genetic determinants of androgenetic alopecia (AGA, male-pattern baldness) that highlight the importance of the androgen pathway in this condition [3], [4]. However, these studies did not identify readily apparent novel pathways that link AGA to other conditions.

AGA is the most common form of hair loss in humans, affecting 80% of men by age 80 [5]. Its etiologic factors are androgen dependency and genetic predisposition [5]. While largely a cosmetic condition, the mechanisms influencing its etiology may also impact upon important medical conditions such as coronary heart disease, metabolic syndrome, and prostate cancer [6], [7].

Prostate cancer is the most frequently diagnosed cancer and ranks second as a cancer killer among men in the United States [8]. Androgens play a key role in stimulation of normal prostate growth and are essential in prostate cancer initiation and progression [9]. The main genetic determinant of AGA is the androgen receptor (*AR*) [3], [4] and prostate cancer susceptibility loci identified through recent GWA studies overlap with androgen receptor binding sites [10], [11] demonstrating a shared etiologic factor in these two conditions. However, while evidence suggests that AGA and prostate cancer are strongly influenced by androgen sensitivity [12], [13], attempts to examine the relationship between AGA and prostate cancer through case-control studies have yielded inconsistent results [7] [14]–[18].

Since previous GWA studies for AGA explained only 13.7% of the variance in this condition [3] we aimed to identify novel determinants of AGA and test their association with common diseases, by undertaking a large-scale meta-analysis of GWA studies involving 12,806 Europeans from seven cohorts included in the Meta-Analysis for Androgenetic Alopecia Novel Determinants (MAAN) consortium.

## Results/Discussion

The current analysis comprised 3,891 cases and 8,915 controls of European ancestry from seven independent studies: Bonn, CoLaus, TwinsUK, Nijmegen Biomedical, 23andMe, Icelandic, and an Australian population based twin study (Table 1). Briefly, we used an extreme discordant case-control design to contrast individuals with early-onset AGA to older individuals without alopecia as assessed by questionnaire, clinical visit or photographs evaluated by a dermatologist, where available. Genome-wide genotyping, using standard platforms, and imputation using the CEU panel of Phase II HapMap were performed. After the quality control criteria were applied, 2,391,230 SNPs remained for genotype-phenotype association analysis.

**Table 1:** Demographic properties of the study subjects in participant studies.

Study	Country of origin	Descent	Study type	Sample size
Bonn	Germany	Germany	Case-control (population based)	582 Cases347 Controls
CoLaus	Switzerland	European	Cohort (community sample)	578 Cases547 Controls
Iceland	Iceland	European	Cohort (population based)	191 Cases198 Controls
Nijmegen	Netherlands	European	Cohort (population based)	73 Cases132 Controls
TwinsUK	UK	UK	Cohort (population based)	162 Cases210 Controls
23andMe	America	European	Cross-sectional	2,167 Cases1,753 Controls
Australian	Australia	European	Cohort (population based)	138 Cases5728 Controls
THISEAS	Grace	European	Case-control	219 Cases297 Controls

The sample size of the current meta-analysis (n = 12,806) was more than fourfold that of the earlier GWA studies of AGA [3], [4]. The genomic inflation factors of the individual studies (Table S1) and overall meta-analysis (λ_gc_ = 1.02) were low, indicating that the observed GWA results were not due to population stratification. The fixed-effect meta-analytic data from all seven cohorts demonstrated a substantial excess of significant associations with AGA at the tail of the QQ plot (Figure S1). We identified a total of 645 SNPs that achieved genome-wide significance (p<5×10^−8^, Table 2, Table S2). The fixed-effect meta-analytic results showed no evidence of substantial heterogeneity across populations (Tables S1 and S2).

**Table 2:** Summary result for the lead SNP from the genome-wide significant loci.

Genetic Variant	Chr.	Position^a^	EA^b^/NEA	EAF	OR (95% CI)	p value	Gene^c^	Number of GW Significant SNPs	*I* ^2^
rs12565727	1	10955669	A/G	0.789	1.33 (1.22–1.45)	9.07×10^−11^	*TARDBP*	8	<0.01
rs9287638	2	239359379	A/C	0.562	1.31 (1.21–1.41)	1.01×10^−12^	*HDAC4*	9	0.10
rs2073963	7	18844399	G/T	0.530	1.29 (1.20–1.38)	1.08×10^−12^	*HDAC9*	30	<0.01
rs6945541	7	68249896	C/T	0.539	1.27 (1.18–1.38)	1.71×10^−9^	*AUTS2*	6	<0.01
rs12373124	17	41279999	T/C	0.438	1.33 (1.21–1.45)	5.07×10^−10^	17q21.31	118	0.24
rs10502861	18	41054146	C/T	0.775	1.28 (1.18–1.39)	2.62×10^−9^	*SETBP1*	16	0.18
rs6047844	20	21985575	T/C	0.460	1.60 (1.49–1.72)	1.71×10^−39^	*PAX1, FOXA2*	277	<0.01
rs2497938	X	66479743	T/C	0.850	2.20 (2.04–2.37)	2.40×10^−91^	*AR*	181	0.32

Abbreviations: Chr., chromosome; EA, effect allele; NEA, non-effect allele; EAF, effect allele frequency; OR, odds ratio; CI, confidence interval.

a -Chromosome position located in reference sequence of Genome Build 36.3.

b -AGA risk increasing allele on forward strand.

c -Gene harboring the SNP or nearest to the SNP.

See Figure S3 for forest plots of these SNPs. See Table S2 for an expanded version of this table that shows all SNPs with p values less than 5×10^−8^.

**Figure 1:**
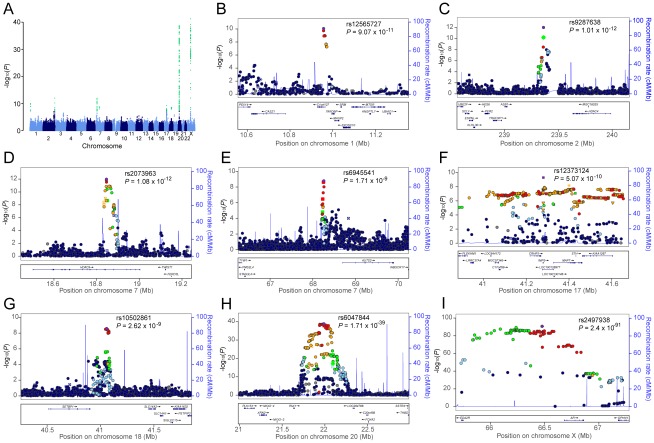
Genome-wide meta-analysis results for AGA in MAAN. (A) Manhattan plot showing the −log_10_ p value of SNPs against their chromosomal positions. The genome-wide significant SNPs are green (p value<5×10^−8^). The points with p value <1×10^−40^ were truncated; the smallest p value was 2.4×10^−91^ at AR gene. (B–I) Regional association plots for eight loci associated with AGA. In each panel, the lead SNP is denoted in purple with its rs ID and association p value. The color of other SNPs indicates the LD with the lead SNP as red (0.8≤*r* ^2^≤1), orange (0.6≤*r* ^2^<0.8), green (0.4≤*r* ^2^<0.6), light blue (0.2≤*r* ^2^<0.4), and dark blue (*r* ^2^<0.2). Estimated recombination rates are in light blue.

Consistent with our previous reports [3], [4], a susceptibility locus for AGA on chromosome 20p11 was confirmed over a ∼253 kb interval with the strongest signal arising at rs6047844 (p = 1.71×10^−39^, odds ratio (OR) = 1.60, 95% confidence interval (CI) = 1.49–1.72) (Figure 1). We also showed confirmed association with AGA at *AR* gene by highly significant signal spanning the gene region (OR = 2.20, 95% CI = 2.04–2.37, p = 2.40×10^−91^ for risk allele T of top SNP rs2497938). Even after removing SNPs in these two established AGA loci, the QQ-plot demonstrated an excess of SNPs associated with AGA (Figure S2).

*FOXA2* (forkhead box A2) is worth noting among the flanking genes at the chromosome 20 locus. Foxa2, the transcription factor encoded by *FOXA2* in mouse, interacts with AR, especially through DNA binding domain, to regulate gene expression [19]. It is expressed in prostate tissue and plays a pivotal role in neuroendocrine prostate tumors, a form of metastatic prostate tumors, by inducing development from androgen-dependent tumors to androgen-independent tumors [20]–[22]. Its expression was associated with the invasive phenotype in the primary prostate cancer [22]. Furthermore, the association of metastatic prostate cancer and AGA through *AR* polymorphisms was previously described in a well-defined case-control study [14].

Multiple SNPs in strong linkage disequilibrium (LD) (*r^2^*>0·6) revealed a newly discovered association interval encompassing several genes on chromosome 17q21.31. The most significant signal of this region was detected for the synonymous (His649His) SNP rs12373124 (*P* = 5.07×10^−10^, OR = 1.33, 95%CI = 1.21–1.45 for risk allele T) at the gene *IMP5* (intramembrane protease 5). However, as the genome-wide significant SNPs are highly-correlated with each other in this region, the most proximal gene is not necessarily the gene functionally affected by causal SNPs. The *MAPT* (microtubule-associated protein tau) gene, which encodes the tau protein, is of particular interest in this region because its expression was detected in hair follicles (p = 9.22×10^−3^) but not in the other tissues examined in our tissue expression analysis (Table S3).

The SNP with the smallest p value, apart from the AGA locus on chromosome 20, (rs9287638 [A]: OR = 1.31, 95% CI = 1.21–1.41, *P* = 1.01×10^−12^), is located on chromosome 2q37, with seven other genome-wide significant SNPs in the same region. These SNPs lie 558 kb downstream of *HDAC4* (histone deacetylase 4). The next locus mapped within *HDAC9* (histone deacetylase 9) on chromosome 7p21.1 (rs2073963 [G]: OR = 1.29, 95% CI = 1.20–1.38, *P* = 1.08×10^−12^). Our expression analysis revealed that *HDAC9* and *HDAC4* were well expressed in hair follicles (p = 6.59×10^−3^ and 2.64×10^−3^ respectively), and *HDAC9* was not expressed in skin or scalp tissues (Table S3).

Thus, two independent signals arose from HDACs (*HDAC4* and *HDAC9*) which act as transcriptional corepressors by deacetylating nucleosomal histones [23]. By interaction with transcription factors ARR19 and CRIF1, HDAC4 plays a critical role in inhibition of AR transactivation [24], [25] and its accumulation coincides with loss of androgen sensitivity in prostate cancer [26]. Furthermore, HDAC9 and HDAC4 share conserved residues and their tissue specific expression pattern overlaps [27]. Very recently, *ZNF652* which has been shown to be involved in transcriptional repression effect of HDACs [28] was identified to be a prostate cancer susceptibility locus [29]. All together, our findings give rise to the possibility that *HDAC4* and *HDAC9* might influence pathogenesis of AGA through dysregulation of the androgen pathway, highlighting a shared etiologic factor in this condition and prostate cancer.

The *AUTS2* (autism susceptibility candidate 2) gene was also identified (lead SNP rs6945541[C]: OR = 1.27, 95%CI = 1.18–1.38, p = 1.71×10^−9^) at the chromosome 7q11.22 locus. The mechanism underlying the association between the *AUTS2* locus and AGA is currently unknown, but its expression profile reveals abundance of transcript in hair, skin, and scalp (p = 2.64×10^−3^, <1.00×10^−3^, and <1.00×10^−3^ respectively, Table S3), but not blood. Additionally, variants at *AUTS2* were recently associated with the regulation of alcohol consumption through a GWA study [30]. The function of *AUTS2* is currently unknown. It was previously associated with autism and mental retardation [30]. How the *AUTS2* variants affect AGA outcomes deserves further investigation.

Remaining novel findings include one locus on chromosome 1 which is near the genes *TARDBP* (TAR DNA binding protein), *PEX14* (peroxisomal biogenesis factor 14), *MASP2* (mannan-binding lectin serine peptidase 2), and *SRM* (spermidine synthase) (lowest p = 9.07×10^−11^ for SNP rs12565727 [A], OR = 1.33, 95%CI = 1.22–1.45) and another locus on chromosome 18q21.1, near the 3′ end of *SETBP1* (SET binding protein 1), of which the most significant was rs10502861 [C] (*P* = 2.62×10^−9^, OR = 1.28, 95%CI = 1.18–1.39). *SETBP1* again demonstrated expression in hair, skin, and scalp (detection p values are 2.64×10^−3^, <1.00×10^−3^, and <1.00×10^−3^ respectively, Table S3), but not blood.

Regional association plots for seven loci associated with AGA are shown in Figure 1. Forest plots of the top SNPs are shown in Figure S3. All these loci were associated with AGA in random-effect results except the locus on chromosome 18 (Table S4).

While any of the above susceptibility loci may impart a small risk, examining the combined effect of these loci in individuals harboring more than one risk allele may improve the ability to identify individuals at high risk of AGA. Using the top SNPs identified in this study from both the 6 novel loci and the 2 confirmed regions on chromosome 20p11 and *AR* gene, we constructed a genotype risk score based on the weighted number of susceptibility alleles in an independent replication sample from 23andMe study. As shown in Table 3, there was an increased risk for AGA across each quartile of the genotypic risk score. For the individuals with a genotype score in the highest quartile, we observed substantially increased odds for AGA (OR = 5.78, 95% CI = 4.86–6.87, p = 1.4×10^−88^), compared to individuals at the lowest quartile.

**Table 3:** Genotype score associated with the risk of androgenetic alopecia.

Genotype score^a^	OR (95% CI)	p value	p value for Trend across all 4 quartiles
Quartile 1	1.00 (Reference)		
Quartile 2	2.20 (1.87–2.59)	6.1×10^−21^	
Quartile 3	3.50 (2.96–4.13)	1.8×10^−49^	
Quartile 4	5.78 (4.86–6.87)	1.4×10^−88^	2.3×10^−101^

Abbreviations: OR, odds ratio; CI, confidence interval.

a -The top SNPs from each genome-wide significant loci as shown in Table 2 are used in risk score analysis.

Reasoning that *AR* gene is replicated as the most substantial genetic component of AGA, we explored the relationship between *AR* gene and other AGA susceptibility loci by several complimentary approaches. First, we examined the effect of *AR* gene on other AGA loci by adding an interaction term in the logistic regression model. We found no evidence for interaction (Table S5), which is consistent with our previous reports [4], [31]. Second, we sought clues of interaction between *AR* gene and other AGA loci mediated by AR binding sites since AR functions as a transcription factor. Although a large number of genes were found to be targets of AR, no AR binding sites have been identified in any of the candidate genes at AGA loci [32], [33]. However, as aforementioned, among these candidate genes, interaction with AR and consequent regulation on an epididymis-specific gene has been shown on *FOXA2* (chromosome 20 locus), and the candidate gene at chromosome 2 locus, *HDAC4*, was reported to act as an inhibitor of AR, especially in prostate cancer cells, suggesting that these two loci may have a role in androgen-dependent pathway.

We identified that the 17q21.31 locus overlaps with a widely replicated locus that is strongly associated with Parkinson's disease [34]–[38]. Interestingly, *MAPT* on this locus contained identical genome-wide significant risk alleles that were shared between AGA (rs2942168 [G], [OR = 1.25, 95% CI = 1.15–1.36, p = 1.95×10^−7^]) and Parkinson's disease (OR = 1.32, 95%CI = 1.23–1.39, p = 1.62×10^−18^) reported in a recently published large GWA study [38]. The lead AGA SNP (rs12373124) at the 17q21.31 locus is in high linkage disequilibrium (*r^2^* = 0·87) with SNPs that have been associated with Parkinson's disease in recent GWA studies [34], [35].

To explore this unexpected relationship between Parkinson's disease and early-onset AGA we next sought to understand whether AGA itself was a risk factor for Parkinson's disease. From among a superset of the early-onset AGA cases and controls using the same definition for AGA as in the meta-analysis, we identified 568 self-reported physician diagnosed Parkinson's disease cases, and 7,664 population controls [39]. This definition of Parkinson's disease has been used previously to replicate genetic loci for this condition in the 23andMe cohort [39]. We found that AGA cases had significantly higher odds of Parkinson's disease (OR = 1.28, 95% CI = 1.06–1.55, p = 8.9×10^−3^). Restricting the analysis to 714 individuals with current age 70 or higher, the association was stronger (OR = 1.94, 95% CI = 1.31–2.88, p = 6.5×10^−4^).

To investigate whether the shared genetic association at 17q21.31 explained the association between Parkinson's disease and AGA, we evaluated a regression model with an additional term for an individual's genotype at rs12185268, which is the variant in this region most strongly associated with Parkinson's disease in the 23andMe study. The OR for association with early-onset AGA and its significance were essentially unchanged (OR = 1.96, 95% CI = 1.32–2.90, p = 6.0×10^−4^). In the individuals in the age 70 or higher group, we also tested the association between Parkinson's disease and AGA stratified by rs12185268 genotypes. The AG and GG genotypes are pooled due to the small proportion of GG homozygotes. There is no essential difference between the odds ratios for individuals with rs12185268 AA genotypes (OR = 1.93, 95% CI = 1.16–3.21, p = 8.7×10^−3^) and AG+GG genotypes (OR = 2.07, 95% CI = 1.09–3.93, p = 2.2×10^−2^), indicating that rs12185268 does not modify the association between Parkinson's disease and early-onset AGA.

We next looked for evidence that any other loci associated with Parkinson's disease were also associated with early-onset AGA, or vice versa. We identified the lead SNPs from 27 loci with p<1.0×10^−5^ for association with AGA, and 31 loci with p<1.0×10^−5^ for association with Parkinson's disease. We tested the AGA loci for association with Parkinson's disease, and the Parkinson's disease loci for association with AGA. The 17q21.31 locus was the only locus demonstrating convincing evidence for association across both phenotypes (Tables S6 and S7).

Parkinson's disease is the second most common neurodegenerative disorder with a prevalence of one percent in individuals that are over 60 years old [40]. Despite the often-reported higher prevalence of Parkinson's disease in men, as compared to women [40], there are no previous reports investigating the relationship between AGA and Parkinson's disease. This novel association between Parkinson's disease and early-onset AGA indicated that there could be a shared genetic or environmental cause for both conditions.

Our data specifically identify genetic variation in the 17q21.31 region as shared genetic risk factors for these two conditions. To date, only drug-induced hair loss has been described in patients having Parkinson's disease after use of dopamine agonist [41], although most of the patients affected by drug-induced hair loss are females [41]. As noted above, a greater incidence of Parkinson's disease has been reported in elderly men than in women [40] and androgen mediated neurotoxicity has been proposed to contribute to the gender bias in Parkinson's disease [42]. Since this association is entirely novel, it is unlikely that our results have arisen due to recall bias. In addition, the AGA cases in 23andMe study had an age of onset less than 40 years old. The mean age at diagnosis of Parkinson's disease is 70.5 years [43]. Therefore it is highly unlikely that Parkinson's disease occurred before AGA, as defined in the 23andMe study. At present we are unaware of any prospective Parkinson's disease cohorts having collected AGA data to further explore this relationship, and our evidence provides rationale to undertake such studies.

Furthermore, the 17q21.31 locus harbors an inversion polymorphism that has previously been described to be under negative selection pressure and has been demonstrated to be associated with decreased fertility in women [44]. We found a genome-wide significant SNP for early-onset AGA, whose risk allele (rs1800547 [A], p = 2.85×10^−8^, OR = 1.27, 95%CI = 1.16–1.39) represents the H1 haplotype of the 17q21.31 inversion. This H1 haplotype is under negative selection pressure in Europeans [45] and Icelandic female carriers of the H1 lineage have fewer children than non-carriers, while men sharing this haplotype have a trend towards decreased fertility [44]. We note that previous studies have identified an association between polycystic ovarian syndrome (PCOS), which is the most common cause of anovulatory infertility in women [46], and early-onset AGA in their male relatives [47], [48]. And increased androgen levels strongly affect both traits. Our findings, therefore, provide rationale to explore the androgen pathway as a possible explanation for the decreased fertility associated with the H1 haplotype in women.

Even though the definition of hair loss and the method of sampling differed between the eight study groups, the associations of five genome-wide significant loci were essentially identical in fixed- and random-effects analysis, suggesting that our results are unlikely to be influenced by heterogeneity.

We are aware that some other diseases, including progressive supranuclear palsy, corticobasal degeneration, frontotemporal dementia and Pick disease are also strongly associated with 17q21.31 region [49], [50]. However it is difficult to test the association between these diseases and AGA. In this study, we focused on the early-onset AGA. The AGA cases we recruited are young. In addition, due to the low prevalence (between 6.4 and 15 per 100,000 [51]–[53]) of these diseases compared to Parkinson's disease, we do not have samples to test the association.

Since 17q21.31 locus has been recognized as a risk factor for Parkinson's disease, it is plausible that an individual's status of Parkinson's disease may have an effect on the association between 17q21.31 locus and early-onset AGA. We undertook an association analysis conditioned on Parkinson's disease in 8,232 individuals for whom both phenotype are available and confirmed that status of Parkinson's disease does not affect the strength of association between AGA and rs12185268, the top variant at 17q21.31 locus in this sample (unconditioned: OR = 0.76, 95% CI = 0.70–0.82, p = 7.9×10^−13^; conditioned: OR = 0.76, 95% CI = 0.70–0.82, p = 7.9×10^−13^). This result was verified within strata of Parkinson's disease: OR is 0.69 in the cases (95% CI = 0.51–0.94, p = 1.8×10^−2^) and 0.76 for individuals without Parkinson's disease (95% CI = 0.71–0.83, p = 1.1×10^−11^). Further, the relationship between the 17q21.31 locus and Parkinson's disease was not affected by conditioning on AGA status although the association with rs12185268 was not significant (unconditioned: OR = 0.99, 95% CI = 0.84–1.15, p = 0.86; conditioned: OR = 1.00, 95% CI = 0.86–1.17, p = 0.97).

Our data demonstrated several aspects of the relationship between AGA and Parkinson's disease: First, variants at 17q21.31 locus including the *MAPT* gene are associated with both risk of Parkinson's disease (p = 2.8×10^−12^) and early onset AGA (p = 9.3×10^−8^) as shown in Table S6 and S7. Second, early onset AGA (age of onset <40 years), is a risk factor for Parkinson's disease (p = 8.9×10^−3^). Third, controlling for variation at rs12185268, the top SNP at 17q21.31 locus, does not eliminate the relationship between AGA and Parkinson's disease. And fourth, the other genetic determinants of AGA, as described through GWAS, do not influence risk of Parkinson's disease and vice versa. Given that the 17q21.31 locus does not fully explain the association between AGA and Parkinson's disease, and that we do not see more broad overlap between susceptibility loci for AGA and Parkinson's disease, it could be that the association between these phenotypes is mediated by an unobserved, shared environmental or genetic risk factor.

The identification of the new associations in this report was driven primarily by augmented power arising from the expanded sample size, which was more than fourfold that of previous GWA analyses [3], [4]. Similar to previous GWA studies [54], this increase in power was associated with a decrease in effect sizes from newly identified loci (top ORs range from 1·27 to 1·33 for six new loci versus OR = 1·60 for the lead SNP on chromosome 20). In this regard, it seems likely that more variants with smaller effect sizes could be uncovered by future larger studies to full describe the allelic architecture of early-onset AGA.

In conclusion, our findings provide fresh insights into the pathogenesis of early-onset AGA. As these newly identified susceptibility loci are also implicated in Parkinson's disease, prostate cancer and fertility, our results highlight the importance of hypothesis-free genetic studies, which allow unexpected genetic relationships between conditions to uncover shared etiologies.

## Materials and Methods

### Ethics Statement

All seven studies were approved by institutional ethics review committees at the relevant organizations, and written informed consent was provided by all participating individuals.

### Study Participants

All the participants for this genome-wide meta-analysis were drawn from seven studies: Bonn (582 cases and 347 controls), CoLasu (578 cases and 547 controls), TwinsUK cohort (162 cases and 210 controls), Nigmegen Biomedical Study (73 cases and 132 controls), 23andMe (2,167 cases and 1,753 controls), Iceland (191 cases and 198 controls), Australian population based twin study (138 unrelated cases and 5728 unrelated controls). A detailed description of all these studies and phenotype definitions used in current study is provided in Text S1.

### Genotyping and Quality Controls

The genotyping platforms, imputation methods and genome wide association methods used in participant studies are provided in Table S1. Extensive quality control thresholds were applied to include common SNPs (minor allele frequency ≥1%) with a high call rate (≥95%) for genotyped SNPs, and imputed SNPs with high quality metrics (variance ratio ≥0.3 for MACH and proper info statistic ≥0.4 for IMPUTE) [55], [56]. In addition, SNPs demonstrating deviation from Hardy Weinberg Equilibrium (p>10^−6^) were excluded. The test statistics for each cohort at each SNP were corrected for their respective genomic inflation factors to avoid inflation of results due to population stratification.

### Meta-Analysis

We carried out a meta-analysis under both fixed- and random-effects models using the inverse-variance method to combine results from each study using an additive genetic model, while correcting for the genomic inflation factor for each study and the overall meta-analysis. To implement this strategy GWAMA software was used for SNPs on autosomes [57]. Based on the data available, association results using pre-imputation SNPs from Bonn, CoLaus, TwinsUK cohort, Nigmegen Biomedical Study, 23andMe and Australian population based twin study were used for meta-analysis on X chromosome through YAMAS program (http://yamas.meb.uni-bonn.de/index.html). Proxy association was applied to gain a higher power. We also tested for evidence of heterogeneity of effects between SNPs and AGA across studies using the Cochran's *Q* statistics and *I^2^* measurement. SNPs with low heterogeneity (*Q* p value>0·10 and *I^2^*<50%) and present in at least three individual studies are reported. In order to test for an inflation of test statistics and the presence of a signal arising from the data for the variants influencing AGA, we constructed quantile-quantile (QQ) plots [58]. Genome-wide significance was set at a p value of 5×10^−8^ [59].

Genome-wide suggestive SNPs (5×10^−8^<p<5×10^−6^, n = 397) that were imputable using the MetaboChip platform were followed-up in an expanded meta-analysis including the THISEAS study [60] (Text S1, 297 controls and 219 cases) and the original seven cohorts. This additional cohort joined the consortium after completion of the main meta-analysis. No additional loci achieved genome-wide significance after inclusion of this cohort.

### Expression Methods

To understand whether the identified SNPs are in close proximity to genes that show differential expression in hair follicles, we performed tissue expression analysis. In brief, total RNA extracted from human hair follicles, skin from temple, scalp, and whole blood were used for array-based gene expression analysis. The differential expression of genes in these tissues was determined by the average signal of identical probes and detection p values (which is significant, if a gene is reliably expressed). Further details are provided in Text S1.

### Genotype Risk Score

In genotype risk score analysis, the association between a genotype risk score based on the weighted number of susceptibility alleles and AGA status was determined. Six novel susceptibility loci, chromosome 20p11 locus and *AR* gene are included. The weights were established using β coefficients for AGA susceptibility from meta-analysis. The resultant genotypic risk score was divided into quartiles and the risk of AGA for each quartile was tested in an additional set of subjects in 23andMe study, which were not included in the original meta-analysis, using the lowest risk quartile as the reference group. We identified 1582 controls and 1765 cases for this analysis, using the same phenotypic definition used in the meta-analysis. The genotype data is complete without missing values based on phasing and imputation. The trend for risk across the quartiles was tested using the non-parametric trend test [61].

### Interaction Analysis

To detect the potential modification effect of *AR* gene on any other AGA loci, an interaction analysis was deployed using top SNPs identified from meta-analysis. Each SNP was coded by the number of AGA risk-increasing allele. The test of interaction is based on the coefficient b_3_ of the interaction term in the logistic regression model Y = b_0_+b_1_ * A+b_2_ * B+b_3_ * AB+e. The Bonferroni correction was applied to address the multiple comparisons problem and the significance level was set to p value<7×10^−3^.

### Association with Other Common Diseases

In order to better understand whether the identified risk loci from the meta-analysis were associated with common diseases, we searched the AGA susceptibility loci using the GWAS Integrator embedded in HuGE Navigator website [62]. This online reference tool collects data from all published genetic studies in humans to facilitate the identification of shared etiologic pathways between diseases. Due to the frequent publication of new GWAS, only the GWAS searched out before June 2011 were used.

To examine the relationship between Parkinson's disease and AGA, we performed a logistic regression of Parkinson's disease status against AGA status, evaluating significance of the AGA term by analysis of deviance using a likelihood ratio test. To control for confounding factors, we included age, age^2^, and the top five principal components derived from genotype data as covariates.

## Supporting Information

Figure S1 Quantile–quantile plot of Meta-Analytic Results. (Genomic Control was applied to each individual cohort prior to meta-analysis and the overall results).
(TIF)

Figure S2 Quantile–quantile plot of Meta-Analytic Results after removing SNPs of AGA locus on chromosome 20 and SNPs of AR locus on chromosome X. (Genomic Control was applied to each individual cohort prior to meta-analysis and the overall results).
(TIF)

Figure S3 Forest Plots of the Lead SNP from Genome-Wide Significant Loci.
(TIF)

Table S1 Genotyping, imputation and statistical analysis used in the GWA studies.
(DOCX)

Table S2 Genome-Wide Significant SNPs in fixed-effect model (p<5×10^−8^).
(DOC)

Table S3 Average Expression Signal for All Genes Shown in Figure 1 across Three Tissue Types and Blood.
(DOC)

Table S4 Summary result for the top SNPs from the genome-wide significant loci using random effect models.
(DOC)

Table S5 Interaction results between top SNPs of *AR* locus and other AGA loci.
(DOC)

Table S6 Association test results for AGA, at Parkinson's disease loci.
(DOC)

Table S7 Association test results for Parkinson's disease, at AGA loci.
(DOC)

Text S1 Description of participant studies and phenotype definition. Methods of tissue expression analysis are also described.
(DOC)
