# Supplementary figures and images for: Six Novel Susceptibility Loci for Early-Onset Androgenetic Alopecia and Their Unexpected Association with Common Diseases
Source: PLoS Genet. 2012 May 31;8(5):e1002746. doi: 10.1371/journal.pgen.1002746 (PMC3364959; doi:10.1371/journal.pgen.1002746)

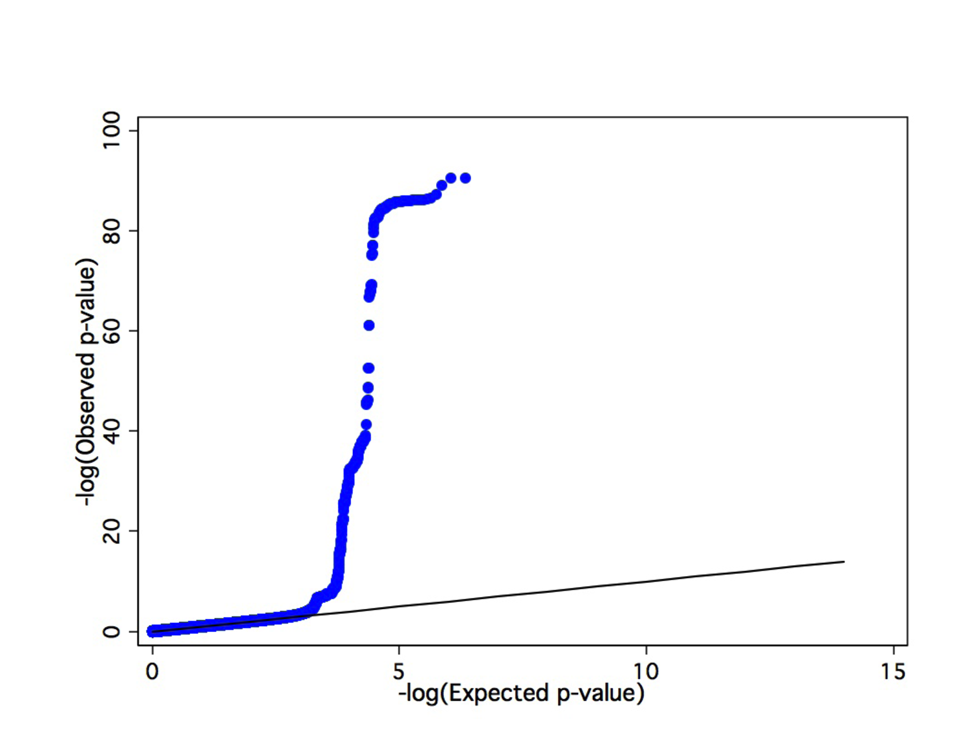

Supplement: Figure S1 — Quantile–quantile plot of Meta-Analytic Results. (Genomic Control was applied to each individual cohort prior to meta-analysis and the overall results). (TIF) [file pgen.1002746.s001.tif]

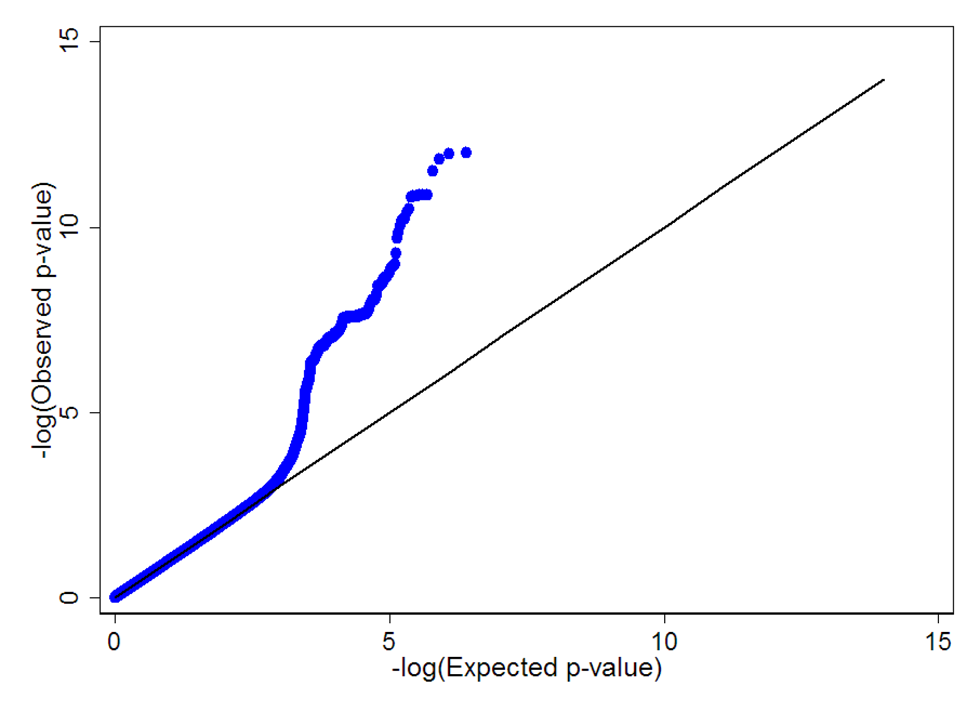

Supplement: Figure S2 — Quantile–quantile plot of Meta-Analytic Results after removing SNPs of AGA locus on chromosome 20 and SNPs of AR locus on chromosome X. (Genomic Control was applied to each individual cohort prior to meta-analysis and the overall results). (TIF) [file pgen.1002746.s002.tif]

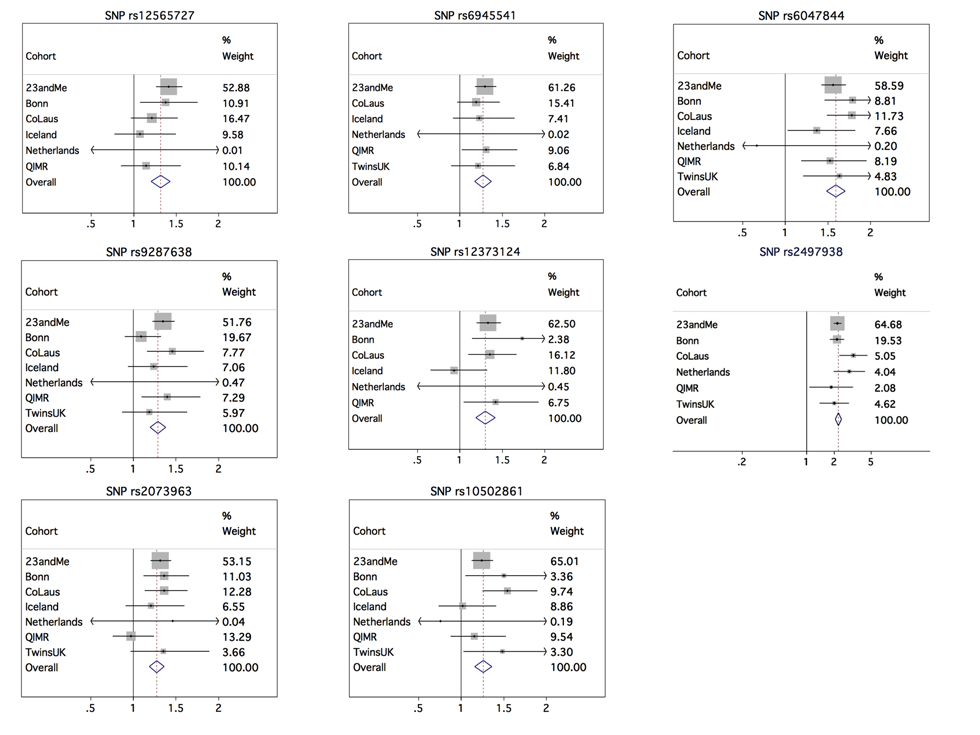

Supplement: Figure S3 — Forest Plots of the Lead SNP from Genome-Wide Significant Loci. (TIF) [file pgen.1002746.s003.tif]
