# Supplementary material for: Six Novel Susceptibility Loci for Early-Onset Androgenetic Alopecia and Their Unexpected Association with Common Diseases
Source: PLoS Genet. 2012 May 31;8(5):e1002746. doi: 10.1371/journal.pgen.1002746 (PMC3364959; doi:10.1371/journal.pgen.1002746)
Supplement: Table S1 — Genotyping, imputation and statistical analysis used in the GWA studies. (DOCX) [file pgen.1002746.s004.docx]

**Table S1** Genotyping, imputation and statistical analysis used in the GWA studies

| **Study** | **Genotyping** | | **Imputation method** | **Genome-wide association analysis** | |
| --- | --- | --- | --- | --- | --- |
|  | platforms/chips | calling algorithm |  | software^a^ | λ_gc_ |
| Bonn | Illumina 317K, 317Kduo, 550K, 610K | Illumina BeadStudio/Genome Studio | IMPUTE[1] | SNPTEST[1] | 1.062 |
| CoLaus | Affymetrix GeneChip Human Mapping 500K | BRLMM algorithm | IMPUTE | PLINK[2] | 1.024 |
| Iceland | Illumina 317k, 317kduo, CNV370k | Illumina BeadStudio | IMPUTE | SNPTEST | 1.022 |
| Nijmegen | Illumina CNV370k | Illumina BeadStudio | IMPUTE | SNPTEST | 1.001 |
| TwinsUK | Illumina 610K | Illuminus | IMPUTE | GWAF^b^[3] | 1.016 |
| 23andMe | Illumina 550+ | Illumina GenomeStudio | MACH | R | 1.033 |
| Australian | Illumina 317K, CNV370K, 610K | Illumina BeadStudio/Genome Studio | MACH | PLINK | 1.016 |
| THISEAS | MetaboChip | GenoSNP | IMPUTE | PLINK | N/A |

^a^-Association analysis on X chromosome was undertaken by using Clayton’s methods[4] implemented in INTERSNP[5]

^b^-The relatedness between siblings were corrected by generalized estimating equations[6] implemented in GWAF

1. Marchini J, Howie B, Myers S, McVean G, Donnelly P (2007) A new multipoint method for genome-wide association studies by imputation of genotypes. Nat Genet 39: 906-913.

2. Purcell S, Neale B, Todd-Brown K, Thomas L, Ferreira MA, et al. (2007) PLINK: a tool set for whole-genome association and population-based linkage analyses. Am J Hum Genet 81: 559-575.

3. Chen MH, Yang Q (2010) GWAF: an R package for genome-wide association analyses with family data. Bioinformatics 26: 580-581.

4. Clayton D (2008) Testing for association on the X chromosome. Biostatistics 9: 593-600.

5. Herold C, Steffens M, Brockschmidt FF, Baur MP, Becker T (2009) INTERSNP: genome-wide interaction analysis guided by a priori information. Bioinformatics 25: 3275-3281.

6. Zeger SL, Liang KY (1986) Longitudinal data analysis for discrete and continuous outcomes. Biometrics 42: 121-130.
