# Supplementary material for: Six Novel Susceptibility Loci for Early-Onset Androgenetic Alopecia and Their Unexpected Association with Common Diseases
Source: PLoS Genet. 2012 May 31;8(5):e1002746. doi: 10.1371/journal.pgen.1002746 (PMC3364959; doi:10.1371/journal.pgen.1002746)
Supplement: Table S2 — Genome-Wide Significant SNPs in fixed-effect model (p<5×10^−8^). (DOC) [file pgen.1002746.s005.doc]

**Table S2** Genome-Wide Significant SNPs in fixed-effect model (p < 5 x 10^-8^)

| **Chr.** | **Position** | **SNP** | **Effect**  **Allele** | **Non Effect**  **Allele** | **Odds Ratio**  **(95% CI)** | **p-value** | ***Q* p-value** | ***I*^2^** |
| --- | --- | --- | --- | --- | --- | --- | --- | --- |
| 1 | 10955414 | rs2003046 | C | A | 1.31 (1.21, 1.42) | 1.99E-10 | 0.41 | 0.02 |
| **1** | **10955669** | **rs12565727** | **G** | **A** | **0.75 (0.69, 0.82)** | **9.07E-11** | **0.43** | **0** |
| 1 | 10957404 | rs9659356 | G | A | 0.77 (0.71, 0.84) | 1.29E-09 | 0.44 | 0 |
| 1 | 10960323 | rs11576658 | T | C | 0.77 (0.71, 0.84) | 1.06E-09 | 0.47 | 0 |
| 1 | 10961063 | rs11121667 | T | C | 0.77 (0.71, 0.84) | 1.17E-09 | 0.44 | 0 |
| 1 | 10962780 | rs7542158 | G | A | 1.29 (1.19, 1.41) | 1.20E-09 | 0.44 | 0 |
| 1 | 10966448 | rs9430158 | G | C | 1.29 (1.18, 1.41) | 2.43E-08 | 0.5 | 0 |
| 1 | 10967416 | rs7547568 | T | A | 1.29 (1.18, 1.42) | 2.20E-08 | 0.51 | 0 |
| 2 | 239357664 | rs4075846 | T | A | 1.26 (1.17, 1.36) | 3.61E-09 | 0.46 | 0 |
| **2** | **239359376** | **rs9287638** | **C** | **A** | **0.77 (0.71, 0.82)** | **1.01E-12** | **0.35** | **0.1** |
| 2 | 239362192 | rs9711321 | T | C | 1.26 (1.18, 1.35) | 6.05E-11 | 0.6 | 0 |
| 2 | 239362352 | rs11683401 | T | C | 1.26 (1.18, 1.35) | 6.02E-11 | 0.6 | 0 |
| 2 | 239387248 | rs9287645 | T | C | 1.23 (1.14, 1.31) | 1.10E-08 | 0.65 | 0 |
| 2 | 239399424 | rs9752491 | G | A | 0.79 (0.73, 0.86) | 3.78E-08 | 0.96 | 0 |
| 2 | 239400560 | rs9751918 | G | A | 1.27 (1.17, 1.38) | 1.71E-08 | 0.94 | 0 |
| 2 | 239401104 | rs9750952 | T | C | 1.26 (1.17, 1.37) | 2.21E-08 | 0.97 | 0 |
| 2 | 239414160 | rs12613833 | T | C | 0.78 (0.72, 0.85) | 2.84E-08 | 0.93 | 0 |
| 7 | 18833368 | rs3852256 | G | A | 1.26 (1.16, 1.37) | 2.16E-08 | 0.1 | 0.46 |
| 7 | 18834556 | rs17349860 | T | C | 1.26 (1.16, 1.36) | 8.66E-09 | 0.14 | 0.39 |
| 7 | 18838216 | rs3852257 | T | C | 1.28 (1.19, 1.37) | 6.75E-11 | 0.15 | 0.37 |
| 7 | 18840910 | rs2016515 | T | G | 0.79 (0.74, 0.85) | 9.75E-10 | 0.18 | 0.33 |
| 7 | 18843424 | rs13238389 | C | A | 0.78 (0.72, 0.84) | 1.46E-11 | 0.22 | 0.28 |
| 7 | 18843932 | rs957960 | C | A | 0.79 (0.74, 0.85) | 2.32E-09 | 0.26 | 0.23 |
| 7 | 18844140 | rs957958 | G | A | 1.29 (1.2, 1.39) | 1.45E-12 | 0.24 | 0.25 |
| **7** | **18844400** | **rs2073963** | **T** | **G** | **0.77 (0.72, 0.83)** | **1.08E-12** | **0.42** | **0** |
| 7 | 18844676 | rs2073964 | G | A | 0.78 (0.72, 0.83) | 3.06E-12 | 0.24 | 0.24 |
| 7 | 18847600 | rs12536836 | T | C | 1.27 (1.18, 1.36) | 4.06E-11 | 0.32 | 0.15 |
| 7 | 18850216 | rs6461386 | G | A | 0.8 (0.75, 0.86) | 2.19E-09 | 0.25 | 0.24 |
| 7 | 18856524 | rs756853 | G | A | 1.27 (1.19, 1.36) | 1.40E-11 | 0.12 | 0.4 |
| 7 | 18857784 | rs13245206 | G | A | 0.78 (0.73, 0.84) | 1.33E-11 | 0.32 | 0.15 |
| 7 | 18861422 | rs10230371 | T | C | 0.79 (0.73, 0.84) | 1.39E-11 | 0.19 | 0.31 |
| 7 | 18862536 | rs2249817 | G | A | 1.27 (1.18, 1.36) | 1.56E-11 | 0.31 | 0.16 |
| 7 | 18864830 | rs6461387 | G | A | 0.79 (0.74, 0.85) | 3.24E-11 | 0.28 | 0.2 |
| 7 | 18869540 | rs756854 | T | C | 1.27 (1.18, 1.36) | 1.39E-10 | 0.29 | 0.19 |
| 7 | 18876756 | rs6461390 | G | A | 1.24 (1.15, 1.33) | 2.44E-08 | 0.4 | 0.03 |
| 7 | 18878314 | rs6951144 | G | C | 1.24 (1.15, 1.34) | 1.95E-08 | 0.39 | 0.04 |
| 7 | 18878482 | rs6951745 | G | A | 0.8 (0.75, 0.87) | 1.32E-08 | 0.49 | 0 |
| 7 | 18879328 | rs6461392 | G | A | 0.8 (0.74, 0.87) | 1.24E-08 | 0.49 | 0 |
| 7 | 18879340 | rs6461393 | T | G | 0.81 (0.75, 0.87) | 2.89E-08 | 0.22 | 0.29 |
| 7 | 18879416 | rs6461394 | G | C | 1.25 (1.16, 1.34) | 9.54E-09 | 0.46 | 0 |
| 7 | 18879904 | rs7794241 | C | A | 0.81 (0.75, 0.87) | 2.67E-08 | 0.29 | 0.18 |
| 7 | 18880776 | rs2286213 | G | C | 1.24 (1.15, 1.34) | 2.60E-08 | 0.29 | 0.18 |
| 7 | 18881952 | rs10237149 | G | A | 0.81 (0.75, 0.87) | 3.73E-08 | 0.24 | 0.26 |
| 7 | 18882084 | rs10237366 | G | C | 0.81 (0.75, 0.87) | 4.56E-08 | 0.39 | 0.04 |
| 7 | 18882192 | rs10237280 | T | C | 1.24 (1.15, 1.33) | 3.49E-08 | 0.39 | 0.04 |
| 7 | 18882226 | rs13233322 | C | A | 0.81 (0.75, 0.87) | 3.81E-08 | 0.23 | 0.27 |
| 7 | 18882400 | rs10486314 | G | A | 1.23 (1.14, 1.33) | 4.96E-08 | 0.15 | 0.38 |
| 7 | 68245712 | rs6947344 | T | C | 1.27 (1.18, 1.38) | 2.14E-09 | 0.96 | 0 |
| 7 | 68248320 | rs4718865 | G | A | 0.79 (0.73, 0.85) | 1.81E-09 | 0.94 | 0 |
| **7** | **68249896** | **rs6945541** | **T** | **C** | **0.78 (0.73, 0.85)** | **1.71E-09** | **0.97** | **0** |
| 7 | 68249920 | rs6965168 | G | A | 0.79 (0.73, 0.85) | 2.41E-09 | 0.95 | 0 |
| 7 | 68250480 | rs1916029 | T | G | 0.79 (0.73, 0.85) | 2.46E-09 | 0.95 | 0 |
| 7 | 68253800 | rs1195228 | G | A | 0.79 (0.72, 0.85) | 9.92E-09 | 0.93 | 0 |
| 17 | 41255568 | rs17762954 | T | C | 0.76 (0.68, 0.83) | 4.87E-08 | 0.44 | 0 |
| 17 | 41279432 | rs12185233 | G | C | 1.28 (1.17, 1.39) | 3.58E-08 | 0.46 | 0 |
| 17 | 41279484 | rs12185235 | T | C | 0.78 (0.72, 0.85) | 2.83E-08 | 0.46 | 0 |
| 17 | 41279712 | rs11079725 | T | C | 1.28 (1.17, 1.39) | 2.66E-08 | 0.46 | 0 |
| 17 | 41279852 | rs12373123 | T | C | 1.28 (1.17, 1.39) | 2.58E-08 | 0.46 | 0 |
| 17 | 41279980 | rs12373142 | G | C | 0.78 (0.72, 0.85) | 2.46E-08 | 0.46 | 0 |
| **17** | **41280000** | **rs12373124** | **T** | **C** | **1.33 (1.21, 1.45)** | **5.07E-10** | **0.25** | **0.24** |
| 17 | 41280116 | rs12373168 | C | A | 0.78 (0.72, 0.85) | 2.38E-08 | 0.46 | 0 |
| 17 | 41280300 | rs17690661 | G | A | 1.28 (1.18, 1.4) | 2.12E-08 | 0.46 | 0 |
| 17 | 41280584 | rs17690679 | G | A | 0.78 (0.72, 0.85) | 2.02E-08 | 0.46 | 0 |
| 17 | 41281384 | rs17769490 | G | A | 1.28 (1.18, 1.4) | 1.84E-08 | 0.45 | 0 |
| 17 | 41283072 | rs17769552 | G | A | 1.29 (1.18, 1.4) | 1.32E-08 | 0.51 | 0 |
| 17 | 41284392 | rs885639 | T | G | 1.28 (1.18, 1.4) | 1.58E-08 | 0.45 | 0 |
| 17 | 41286904 | rs2873269 | T | C | 0.78 (0.71, 0.85) | 1.68E-08 | 0.45 | 0 |
| 17 | 41288960 | rs10445368 | T | C | 0.78 (0.71, 0.85) | 2.05E-08 | 0.43 | 0 |
| 17 | 41333624 | rs1864325 | T | C | 0.79 (0.73, 0.86) | 4.96E-08 | 0.38 | 0.07 |
| 17 | 41381748 | rs242559 | C | A | 0.79 (0.73, 0.86) | 3.53E-08 | 0.6 | 0 |
| 17 | 41388632 | rs17571718 | T | C | 1.27 (1.16, 1.38) | 4.51E-08 | 0.42 | 0.01 |
| 17 | 41388780 | rs17571739 | T | C | 1.27 (1.17, 1.38) | 4.15E-08 | 0.42 | 0.01 |
| 17 | 41389668 | rs17571781 | T | C | 1.27 (1.17, 1.38) | 3.98E-08 | 0.41 | 0.01 |
| 17 | 41390696 | rs17571809 | G | A | 0.79 (0.72, 0.86) | 3.93E-08 | 0.42 | 0.01 |
| 17 | 41391204 | rs17650771 | G | A | 0.78 (0.71, 0.85) | 4.53E-08 | 0.35 | 0.11 |
| 17 | 41391544 | rs17571857 | G | A | 0.79 (0.72, 0.86) | 3.08E-08 | 0.41 | 0.02 |
| 17 | 41392624 | rs17650818 | T | C | 1.27 (1.17, 1.38) | 2.96E-08 | 0.41 | 0.02 |
| 17 | 41393328 | rs17650842 | G | A | 0.79 (0.72, 0.86) | 2.82E-08 | 0.41 | 0.02 |
| 17 | 41394844 | rs17650860 | G | A | 1.27 (1.17, 1.38) | 2.77E-08 | 0.41 | 0.02 |
| 17 | 41395352 | rs17650872 | T | G | 0.79 (0.72, 0.85) | 2.55E-08 | 0.4 | 0.03 |
| 17 | 41395528 | rs17650901 | G | A | 0.79 (0.72, 0.86) | 2.75E-08 | 0.41 | 0.02 |
| 17 | 41400056 | rs17650973 | T | A | 0.79 (0.72, 0.86) | 2.71E-08 | 0.42 | 0.01 |
| 17 | 41400344 | rs17650991 | C | A | 0.77 (0.71, 0.84) | 6.85E-09 | 0.45 | 0 |
| 17 | 41400820 | rs17572147 | G | A | 0.79 (0.72, 0.86) | 2.71E-08 | 0.42 | 0.01 |
| 17 | 41401808 | rs17572169 | T | C | 0.79 (0.72, 0.86) | 2.71E-08 | 0.42 | 0.01 |
| 17 | 41405384 | rs17651093 | G | A | 1.27 (1.17, 1.38) | 2.65E-08 | 0.42 | 0.01 |
| 17 | 41405704 | rs17572248 | G | A | 0.79 (0.72, 0.86) | 2.69E-08 | 0.42 | 0.01 |
| 17 | 41406176 | rs17651134 | G | A | 1.27 (1.17, 1.38) | 2.77E-08 | 0.42 | 0 |
| 17 | 41407680 | rs1800547 | G | A | 0.79 (0.72, 0.86) | 2.85E-08 | 0.42 | 0 |
| 17 | 41407760 | rs17651213 | G | A | 1.27 (1.17, 1.38) | 3.35E-08 | 0.44 | 0 |
| 17 | 41407844 | rs17572361 | T | C | 1.27 (1.17, 1.38) | 2.84E-08 | 0.42 | 0 |
| 17 | 41408120 | rs17651243 | G | A | 1.27 (1.17, 1.38) | 2.81E-08 | 0.42 | 0 |
| 17 | 41409284 | rs2217394 | G | A | 0.79 (0.72, 0.86) | 2.78E-08 | 0.42 | 0 |
| 17 | 41410072 | rs17651285 | G | A | 1.27 (1.17, 1.38) | 2.78E-08 | 0.42 | 0 |
| 17 | 41410224 | rs17572467 | T | C | 1.27 (1.17, 1.38) | 2.77E-08 | 0.42 | 0 |
| 17 | 41410432 | rs17572495 | T | G | 1.27 (1.17, 1.38) | 2.77E-08 | 0.42 | 0 |
| 17 | 41411484 | rs754512 | T | A | 0.79 (0.72, 0.86) | 2.77E-08 | 0.42 | 0 |
| 17 | 41412668 | rs1981998 | G | A | 1.27 (1.17, 1.38) | 2.34E-08 | 0.42 | 0 |
| 17 | 41413840 | rs17572613 | G | A | 0.79 (0.72, 0.85) | 2.33E-08 | 0.42 | 0 |
| 17 | 41414464 | rs17572627 | T | A | 1.27 (1.17, 1.38) | 2.31E-08 | 0.42 | 0 |
| 17 | 41414696 | rs17651483 | C | A | 1.27 (1.17, 1.38) | 2.30E-08 | 0.42 | 0.01 |
| 17 | 41417116 | rs17651549 | T | C | 0.79 (0.72, 0.85) | 2.28E-08 | 0.42 | 0 |
| 17 | 41418976 | rs1529534 | G | A | 0.79 (0.72, 0.85) | 2.24E-08 | 0.42 | 0.01 |
| 17 | 41419232 | rs17572795 | G | A | 1.27 (1.17, 1.38) | 2.20E-08 | 0.42 | 0.01 |
| 17 | 41419400 | rs17572823 | T | C | 1.27 (1.17, 1.38) | 2.21E-08 | 0.42 | 0.01 |
| 17 | 41419604 | rs17572851 | G | A | 0.79 (0.72, 0.85) | 2.21E-08 | 0.42 | 0.01 |
| 17 | 41420044 | rs17572893 | G | A | 1.27 (1.17, 1.38) | 2.22E-08 | 0.42 | 0.01 |
| 17 | 41420596 | rs1529535 | C | A | 0.79 (0.72, 0.85) | 2.22E-08 | 0.42 | 0.01 |
| 17 | 41421248 | rs10445371 | G | A | 1.27 (1.17, 1.38) | 2.42E-08 | 0.42 | 0 |
| 17 | 41533808 | rs17660464 | C | A | 1.29 (1.18, 1.41) | 2.60E-08 | 0.41 | 0.01 |
| 17 | 41545156 | rs17660847 | T | C | 0.77 (0.7, 0.84) | 2.34E-08 | 0.39 | 0.04 |
| 17 | 41548344 | rs4630591 | T | C | 0.77 (0.7, 0.84) | 3.47E-08 | 0.34 | 0.12 |
| 17 | 41577380 | rs2696590 | G | C | 1.3 (1.19, 1.42) | 2.61E-08 | 0.39 | 0.04 |
| 17 | 41583944 | rs1918793 | T | C | 0.78 (0.71, 0.85) | 2.43E-08 | 0.35 | 0.1 |
| 17 | 41584304 | rs1918792 | G | A | 1.29 (1.18, 1.41) | 2.62E-08 | 0.43 | 0 |
| 17 | 41584384 | rs1918791 | G | C | 0.78 (0.71, 0.85) | 2.62E-08 | 0.43 | 0 |
| 17 | 41584396 | rs1918790 | T | C | 0.78 (0.71, 0.85) | 2.62E-08 | 0.43 | 0 |
| 17 | 41584548 | rs2696576 | G | A | 0.78 (0.71, 0.85) | 2.63E-08 | 0.43 | 0 |
| 17 | 41584600 | rs2696575 | G | A | 0.78 (0.71, 0.85) | 2.63E-08 | 0.43 | 0 |
| 17 | 41585140 | rs2532303 | T | C | 1.29 (1.18, 1.41) | 2.63E-08 | 0.43 | 0 |
| 17 | 41585192 | rs2532302 | T | C | 1.29 (1.18, 1.41) | 2.63E-08 | 0.43 | 0 |
| 17 | 41585872 | rs2696574 | T | C | 0.77 (0.7, 0.84) | 2.49E-08 | 0.41 | 0.02 |
| 17 | 41586424 | rs2696573 | T | C | 0.78 (0.71, 0.85) | 2.66E-08 | 0.43 | 0 |
| 17 | 41587072 | rs2532298 | G | A | 1.29 (1.18, 1.41) | 2.65E-08 | 0.43 | 0 |
| 17 | 41587104 | rs2532297 | G | A | 1.29 (1.18, 1.41) | 2.65E-08 | 0.43 | 0 |
| 17 | 41587392 | rs2696572 | T | A | 0.78 (0.71, 0.85) | 2.66E-08 | 0.43 | 0 |
| 17 | 41587604 | rs2532296 | T | C | 1.29 (1.18, 1.41) | 2.66E-08 | 0.43 | 0 |
| 17 | 41588736 | rs2109092 | G | A | 1.29 (1.18, 1.41) | 2.66E-08 | 0.43 | 0 |
| 17 | 41588768 | rs1534456 | T | C | 1.3 (1.18, 1.42) | 2.45E-08 | 0.5 | 0 |
| 17 | 41589552 | rs17662235 | T | C | 1.29 (1.18, 1.41) | 2.66E-08 | 0.43 | 0 |
| 17 | 41589588 | rs17585214 | T | C | 0.78 (0.71, 0.85) | 2.67E-08 | 0.43 | 0 |
| 17 | 41589836 | rs1528074 | G | A | 0.77 (0.71, 0.84) | 2.46E-08 | 0.5 | 0 |
| 17 | 41590304 | rs1406068 | T | C | 0.78 (0.71, 0.85) | 2.68E-08 | 0.43 | 0 |
| 17 | 41592504 | rs1528072 | C | A | 1.29 (1.18, 1.41) | 2.98E-08 | 0.43 | 0 |
| 17 | 41592844 | rs2532292 | T | A | 0.78 (0.71, 0.85) | 2.69E-08 | 0.43 | 0 |
| 17 | 41593148 | rs2696571 | G | C | 1.29 (1.18, 1.41) | 2.71E-08 | 0.43 | 0 |
| 17 | 41594200 | rs2532291 | G | A | 1.29 (1.18, 1.41) | 2.75E-08 | 0.43 | 0 |
| 17 | 41594268 | rs2532290 | G | A | 1.29 (1.18, 1.41) | 2.76E-08 | 0.43 | 0 |
| 17 | 41594716 | rs17662403 | T | C | 1.29 (1.18, 1.41) | 2.76E-08 | 0.43 | 0 |
| 17 | 41594744 | rs17585426 | T | C | 1.29 (1.18, 1.41) | 2.77E-08 | 0.43 | 0 |
| 17 | 41595736 | rs2532288 | T | C | 0.78 (0.71, 0.85) | 2.78E-08 | 0.43 | 0 |
| 17 | 41595884 | rs1918789 | T | C | 1.29 (1.18, 1.41) | 2.79E-08 | 0.43 | 0 |
| 17 | 41596764 | rs2141299 | C | A | 1.29 (1.18, 1.41) | 2.79E-08 | 0.43 | 0 |
| 17 | 41597080 | rs2696567 | G | C | 0.78 (0.71, 0.85) | 2.79E-08 | 0.43 | 0 |
| 17 | 41597440 | rs2532286 | T | C | 1.29 (1.18, 1.41) | 2.80E-08 | 0.43 | 0 |
| 17 | 41598956 | rs4792843 | G | A | 0.78 (0.71, 0.85) | 2.80E-08 | 0.43 | 0 |
| 17 | 41599756 | rs17585608 | T | C | 0.78 (0.71, 0.85) | 2.80E-08 | 0.43 | 0 |
| 17 | 41600176 | rs2696684 | G | A | 1.29 (1.18, 1.41) | 2.80E-08 | 0.43 | 0 |
| 17 | 41600360 | rs17585644 | T | C | 1.29 (1.18, 1.41) | 2.82E-08 | 0.43 | 0 |
| 17 | 41600672 | rs2532282 | G | C | 0.77 (0.71, 0.84) | 2.62E-08 | 0.41 | 0.01 |
| 17 | 41600704 | rs2696657 | T | G | 1.29 (1.18, 1.41) | 2.86E-08 | 0.43 | 0 |
| 17 | 41600948 | rs2532281 | T | G | 0.77 (0.71, 0.84) | 2.62E-08 | 0.41 | 0.01 |
| 17 | 41601136 | rs2532280 | G | A | 1.3 (1.18, 1.42) | 2.64E-08 | 0.41 | 0.01 |
| 17 | 41601988 | rs2696660 | G | A | 0.78 (0.71, 0.85) | 2.89E-08 | 0.43 | 0 |
| 17 | 41602184 | rs2532278 | C | A | 1.29 (1.18, 1.41) | 2.89E-08 | 0.43 | 0 |
| 17 | 41602304 | rs2532277 | T | C | 1.3 (1.18, 1.42) | 2.63E-08 | 0.41 | 0.01 |
| 17 | 41602400 | rs2532276 | C | A | 1.29 (1.18, 1.41) | 2.78E-08 | 0.43 | 0 |
| 17 | 41602776 | rs2532275 | G | A | 1.29 (1.18, 1.41) | 2.88E-08 | 0.43 | 0 |
| 17 | 41602940 | rs2532274 | G | A | 0.78 (0.71, 0.85) | 2.44E-08 | 0.37 | 0.08 |
| 17 | 41603092 | rs2532273 | T | C | 0.78 (0.71, 0.85) | 2.88E-08 | 0.43 | 0 |
| 17 | 41603820 | rs2532271 | G | A | 1.29 (1.18, 1.41) | 2.90E-08 | 0.43 | 0 |
| 17 | 41604544 | rs1881193 | T | C | 1.29 (1.18, 1.41) | 2.89E-08 | 0.43 | 0 |
| 17 | 41604592 | rs1881194 | G | A | 1.29 (1.18, 1.41) | 2.90E-08 | 0.43 | 0 |
| 17 | 41604872 | rs17662889 | C | A | 0.77 (0.7, 0.84) | 2.06E-08 | 0.5 | 0 |
| 17 | 41605884 | rs2532269 | T | C | 1.3 (1.18, 1.42) | 2.73E-08 | 0.39 | 0.04 |
| 17 | 41606248 | rs2532268 | G | C | 1.3 (1.18, 1.42) | 2.60E-08 | 0.41 | 0.02 |
| 17 | 41612072 | rs2532253 | G | A | 1.3 (1.18, 1.42) | 2.64E-08 | 0.41 | 0.02 |
| 17 | 41615316 | rs2732585 | C | A | 0.77 (0.71, 0.85) | 2.71E-08 | 0.41 | 0.01 |
| 17 | 41615568 | rs2696700 | G | A | 1.31 (1.19, 1.44) | 2.76E-08 | 0.49 | 0 |
| 17 | 41621256 | rs2696709 | G | C | 1.29 (1.18, 1.42) | 2.82E-08 | 0.41 | 0.01 |
| 17 | 41634440 | rs2696446 | G | A | 1.29 (1.18, 1.42) | 3.46E-08 | 0.42 | 0 |
| 18 | 41043488 | rs8083006 | T | C | 0.78 (0.72, 0.85) | 3.25E-09 | 0.21 | 0.29 |
| **18** | **41054144** | **rs10502861** | **T** | **C** | **0.78 (0.72, 0.85)** | **2.62E-09** | **0.29** | **0.18** |
| 18 | 41057328 | rs1381556 | T | C | 1.28 (1.18, 1.38) | 3.70E-09 | 0.3 | 0.18 |
| 18 | 41058136 | rs1350905 | G | A | 0.78 (0.72, 0.85) | 3.74E-09 | 0.3 | 0.17 |
| 18 | 41058396 | rs1350904 | G | A | 1.28 (1.18, 1.38) | 3.74E-09 | 0.3 | 0.17 |
| 18 | 41058524 | rs12606816 | G | A | 0.78 (0.72, 0.85) | 3.85E-09 | 0.31 | 0.16 |
| 18 | 41061932 | rs12959797 | G | A | 1.28 (1.18, 1.38) | 3.40E-09 | 0.3 | 0.16 |
| 18 | 41064384 | rs8098865 | G | A | 0.78 (0.72, 0.85) | 3.85E-09 | 0.3 | 0.17 |
| 18 | 41066892 | rs1903647 | G | A | 0.79 (0.73, 0.86) | 9.84E-09 | 0.26 | 0.22 |
| 18 | 41066976 | rs1903646 | T | A | 1.27 (1.17, 1.37) | 9.40E-09 | 0.26 | 0.22 |
| 18 | 41068152 | rs8085664 | C | A | 1.27 (1.17, 1.37) | 1.07E-08 | 0.28 | 0.19 |
| 18 | 41068488 | rs7230186 | G | C | 0.79 (0.73, 0.86) | 9.14E-09 | 0.27 | 0.2 |
| 18 | 41072524 | rs11082435 | T | C | 1.27 (1.17, 1.37) | 8.27E-09 | 0.29 | 0.19 |
| 18 | 41075456 | rs11664621 | T | C | 0.79 (0.73, 0.85) | 9.36E-09 | 0.23 | 0.26 |
| 18 | 41075696 | rs7240906 | T | C | 1.27 (1.17, 1.38) | 8.69E-09 | 0.23 | 0.26 |
| 18 | 41075964 | rs6507604 | T | C | 1.27 (1.17, 1.38) | 6.06E-09 | 0.19 | 0.31 |
| 20 | 21714736 | rs972014 | T | C | 1.51 (1.34, 1.71) | 1.50E-11 | 0.17 | 0.34 |
| 20 | 21718656 | rs1476396 | G | C | 1.39 (1.29, 1.5) | 4.76E-17 | 0.23 | 0.27 |
| 20 | 21719760 | rs2208309 | G | A | 0.72 (0.67, 0.78) | 4.63E-17 | 0.23 | 0.27 |
| 20 | 21719844 | rs2208310 | T | G | 0.66 (0.58, 0.75) | 4.31E-10 | 0.14 | 0.39 |
| 20 | 21719908 | rs2224366 | G | C | 0.66 (0.58, 0.75) | 4.29E-10 | 0.14 | 0.39 |
| 20 | 21723784 | rs1476398 | G | A | 1.5 (1.31, 1.71) | 6.15E-09 | 0.1 | 0.43 |
| 20 | 21724736 | rs2208312 | T | C | 0.74 (0.69, 0.8) | 5.15E-14 | 0.19 | 0.33 |
| 20 | 21724876 | rs2208314 | C | A | 1.35 (1.25, 1.45) | 4.65E-14 | 0.19 | 0.33 |
| 20 | 21725916 | rs2876622 | G | A | 1.39 (1.29, 1.5) | 4.32E-17 | 0.24 | 0.26 |
| 20 | 21728196 | rs6047632 | T | C | 1.35 (1.25, 1.45) | 4.83E-14 | 0.17 | 0.35 |
| 20 | 21728904 | rs6082512 | T | C | 0.72 (0.67, 0.78) | 5.94E-17 | 0.2 | 0.32 |
| 20 | 21728972 | rs6082513 | C | A | 1.39 (1.28, 1.49) | 6.15E-17 | 0.2 | 0.32 |
| 20 | 21729624 | rs6047635 | T | G | 0.66 (0.59, 0.75) | 1.57E-11 | 0.23 | 0.27 |
| 20 | 21732132 | rs873137 | C | A | 1.53 (1.35, 1.73) | 1.08E-11 | 0.28 | 0.2 |
| 20 | 21733640 | rs6137444 | T | C | 1.41 (1.31, 1.52) | 1.10E-18 | 0.21 | 0.3 |
| 20 | 21736176 | rs1540930 | G | C | 1.53 (1.33, 1.76) | 2.72E-09 | 0.12 | 0.41 |
| 20 | 21736460 | rs6075840 | T | A | 1.53 (1.35, 1.72) | 1.24E-11 | 0.27 | 0.21 |
| 20 | 21737804 | rs6137445 | G | A | 1.53 (1.35, 1.73) | 1.13E-11 | 0.26 | 0.22 |
| 20 | 21738076 | rs4815066 | C | A | 1.54 (1.34, 1.77) | 2.06E-09 | 0.12 | 0.41 |
| 20 | 21738860 | rs6137446 | G | A | 1.53 (1.36, 1.73) | 8.77E-12 | 0.27 | 0.21 |
| 20 | 21739008 | rs6137448 | T | A | 1.41 (1.31, 1.53) | 9.93E-19 | 0.18 | 0.34 |
| 20 | 21739854 | rs6047641 | T | G | 0.68 (0.6, 0.77) | 3.97E-09 | 0.97 | 0 |
| 20 | 21741400 | rs6047643 | T | A | 1.54 (1.34, 1.77) | 1.78E-09 | 0.12 | 0.41 |
| 20 | 21744856 | rs2180700 | T | G | 1.42 (1.31, 1.53) | 8.25E-19 | 0.19 | 0.33 |
| 20 | 21745348 | rs1540931 | C | A | 1.5 (1.33, 1.7) | 1.06E-10 | 0.12 | 0.4 |
| 20 | 21749468 | rs6035959 | G | A | 0.63 (0.56, 0.71) | 2.63E-14 | 0.17 | 0.34 |
| 20 | 21749500 | rs6082519 | T | A | 0.69 (0.64, 0.74) | 3.15E-21 | 0.28 | 0.2 |
| 20 | 21751314 | rs16983003 | T | A | 1.3 (1.2, 1.42) | 1.13E-09 | 0.14 | 0.4 |
| 20 | 21751580 | rs4281974 | G | A | 0.7 (0.64, 0.75) | 5.11E-20 | 0.29 | 0.19 |
| 20 | 21760118 | rs6082520 | T | C | 1.46 (1.35, 1.57) | 4.73E-21 | 0.27 | 0.21 |
| 20 | 21762036 | rs2224261 | T | C | 0.69 (0.64, 0.74) | 4.98E-21 | 0.27 | 0.21 |
| 20 | 21763192 | rs6113393 | T | C | 1.51 (1.4, 1.63) | 1.65E-26 | 0.58 | 0 |
| 20 | 21766308 | rs1535199 | G | A | 0.66 (0.61, 0.71) | 1.83E-26 | 0.58 | 0 |
| 20 | 21769148 | rs6113394 | T | G | 0.66 (0.61, 0.71) | 1.88E-26 | 0.58 | 0 |
| 20 | 21769216 | rs6106421 | T | C | 1.51 (1.4, 1.63) | 1.89E-26 | 0.58 | 0 |
| 20 | 21769262 | rs6113395 | G | A | 1.51 (1.4, 1.63) | 1.89E-26 | 0.58 | 0 |
| 20 | 21769910 | rs6047663 | T | C | 1.51 (1.4, 1.63) | 1.97E-26 | 0.58 | 0 |
| 20 | 21772164 | rs4405823 | T | A | 0.66 (0.62, 0.71) | 1.99E-26 | 0.58 | 0 |
| 20 | 21772282 | rs8122229 | G | A | 1.51 (1.4, 1.63) | 2.05E-26 | 0.58 | 0 |
| 20 | 21778508 | rs2328647 | G | A | 1.5 (1.4, 1.62) | 3.83E-26 | 0.6 | 0 |
| 20 | 21781736 | rs2064773 | G | A | 1.39 (1.29, 1.5) | 2.40E-16 | 0.43 | 0 |
| 20 | 21781826 | rs2144882 | T | C | 1.87 (1.53, 2.3) | 1.89E-09 | 0.53 | 0 |
| 20 | 21790888 | rs6047676 | T | C | 1.51 (1.4, 1.63) | 1.78E-26 | 0.58 | 0 |
| 20 | 21792630 | rs1014883 | G | A | 1.51 (1.4, 1.63) | 1.80E-26 | 0.58 | 0 |
| 20 | 21792694 | rs1014884 | T | C | 0.66 (0.62, 0.72) | 2.11E-26 | 0.59 | 0 |
| 20 | 21792828 | rs1014885 | T | C | 1.51 (1.4, 1.63) | 1.05E-26 | 0.56 | 0 |
| 20 | 21795480 | rs6047677 | G | C | 0.66 (0.62, 0.72) | 2.04E-26 | 0.58 | 0 |
| 20 | 21798786 | rs12479719 | G | A | 0.74 (0.69, 0.8) | 2.80E-13 | 0.47 | 0 |
| 20 | 21798850 | rs997078 | T | A | 0.66 (0.62, 0.72) | 2.21E-26 | 0.58 | 0 |
| 20 | 21800048 | rs987050 | C | A | 1.51 (1.4, 1.62) | 2.92E-26 | 0.53 | 0 |
| 20 | 21801192 | rs6047683 | C | A | 1.51 (1.4, 1.62) | 2.39E-26 | 0.58 | 0 |
| 20 | 21801764 | rs6047684 | G | A | 0.66 (0.62, 0.72) | 2.39E-26 | 0.58 | 0 |
| 20 | 21803904 | rs6047685 | T | A | 1.51 (1.4, 1.62) | 2.39E-26 | 0.58 | 0 |
| 20 | 21804148 | rs6035971 | T | A | 1.51 (1.4, 1.62) | 2.40E-26 | 0.58 | 0 |
| 20 | 21811824 | rs6113404 | T | C | 0.65 (0.61, 0.7) | 4.21E-30 | 0.13 | 0.39 |
| 20 | 21814152 | rs6075852 | G | A | 1.54 (1.44, 1.65) | 7.41E-33 | 0.33 | 0.14 |
| 20 | 21814376 | rs6075854 | G | A | 0.65 (0.61, 0.7) | 3.04E-30 | 0.15 | 0.37 |
| 20 | 21814548 | rs6075855 | T | C | 0.65 (0.61, 0.7) | 4.64E-30 | 0.13 | 0.39 |
| 20 | 21823140 | rs6035978 | T | C | 0.65 (0.6, 0.7) | 2.47E-30 | 0.14 | 0.37 |
| 20 | 21823172 | rs6035979 | T | G | 1.54 (1.43, 1.65) | 2.42E-30 | 0.14 | 0.37 |
| 20 | 21823656 | rs6047705 | T | C | 0.65 (0.6, 0.7) | 2.34E-30 | 0.14 | 0.37 |
| 20 | 21825020 | rs4815081 | G | A | 0.65 (0.6, 0.7) | 2.17E-30 | 0.14 | 0.37 |
| 20 | 21828044 | rs1998076 | G | A | 1.54 (1.43, 1.65) | 1.89E-30 | 0.14 | 0.38 |
| 20 | 21832692 | rs6137473 | G | A | 0.66 (0.61, 0.71) | 8.18E-28 | 0.24 | 0.25 |
| 20 | 21833312 | rs4544515 | T | C | 1.56 (1.45, 1.67) | 1.74E-33 | 0.1 | 0.43 |
| 20 | 21833500 | rs1884588 | T | C | 1.54 (1.43, 1.65) | 2.78E-33 | 0.22 | 0.27 |
| 20 | 21833620 | rs1884589 | C | A | 1.54 (1.43, 1.65) | 2.94E-33 | 0.22 | 0.27 |
| 20 | 21834168 | rs6113415 | G | A | 0.65 (0.61, 0.7) | 3.04E-33 | 0.22 | 0.27 |
| 20 | 21834362 | rs6113416 | T | C | 1.54 (1.43, 1.65) | 3.35E-33 | 0.22 | 0.27 |
| 20 | 21835266 | rs6047715 | T | A | 1.54 (1.43, 1.65) | 3.47E-33 | 0.22 | 0.27 |
| 20 | 21837318 | rs6113418 | T | A | 0.65 (0.61, 0.7) | 3.61E-33 | 0.22 | 0.27 |
| 20 | 21838690 | rs1884592 | T | C | 1.53 (1.43, 1.64) | 3.99E-33 | 0.26 | 0.23 |
| 20 | 21838816 | rs1884593 | C | A | 0.65 (0.61, 0.7) | 3.75E-33 | 0.22 | 0.27 |
| 20 | 21839164 | rs6082558 | G | A | 0.65 (0.61, 0.7) | 3.75E-33 | 0.22 | 0.27 |
| 20 | 21847028 | rs6075858 | G | A | 0.66 (0.61, 0.71) | 3.13E-27 | 0.17 | 0.35 |
| 20 | 21847248 | rs6047732 | G | C | 0.65 (0.61, 0.7) | 3.70E-33 | 0.22 | 0.27 |
| 20 | 21847708 | rs2208054 | G | A | 1.53 (1.43, 1.65) | 3.64E-33 | 0.22 | 0.27 |
| 20 | 21847864 | rs2208055 | G | A | 1.53 (1.43, 1.65) | 3.60E-33 | 0.22 | 0.27 |
| 20 | 21848116 | rs4390829 | G | A | 1.54 (1.43, 1.65) | 2.74E-33 | 0.22 | 0.27 |
| 20 | 21849124 | rs3818179 | T | C | 0.65 (0.61, 0.7) | 4.03E-33 | 0.21 | 0.29 |
| 20 | 21849398 | rs3818182 | C | A | 1.53 (1.43, 1.64) | 4.02E-33 | 0.21 | 0.29 |
| 20 | 21853732 | rs6515191 | T | A | 0.65 (0.61, 0.7) | 4.43E-33 | 0.23 | 0.26 |
| 20 | 21854780 | rs6113424 | G | A | 0.65 (0.61, 0.7) | 3.34E-33 | 0.23 | 0.27 |
| 20 | 21855562 | rs6113425 | G | A | 0.65 (0.61, 0.7) | 2.80E-33 | 0.23 | 0.27 |
| 20 | 21856332 | rs2281526 | G | C | 1.53 (1.43, 1.64) | 2.52E-33 | 0.23 | 0.27 |
| 20 | 21862194 | rs927059 | T | C | 0.65 (0.61, 0.7) | 7.56E-33 | 0.24 | 0.24 |
| 20 | 21864356 | rs6113431 | T | A | 1.53 (1.43, 1.64) | 2.57E-33 | 0.23 | 0.26 |
| 20 | 21865392 | rs2024885 | G | A | 1.53 (1.43, 1.64) | 2.90E-33 | 0.24 | 0.25 |
| 20 | 21866106 | rs2328680 | T | C | 1.53 (1.43, 1.64) | 2.52E-33 | 0.23 | 0.26 |
| 20 | 21866212 | rs2328681 | T | C | 0.65 (0.61, 0.7) | 2.50E-33 | 0.23 | 0.26 |
| 20 | 21866288 | rs2328682 | G | A | 0.65 (0.61, 0.7) | 2.47E-33 | 0.23 | 0.26 |
| 20 | 21866310 | rs1007169 | T | G | 1.53 (1.43, 1.64) | 2.39E-33 | 0.24 | 0.25 |
| 20 | 21866428 | rs2208050 | G | A | 0.65 (0.61, 0.7) | 2.03E-33 | 0.24 | 0.25 |
| 20 | 21866472 | rs2208051 | G | A | 1.54 (1.43, 1.65) | 1.55E-33 | 0.24 | 0.25 |
| 20 | 21866642 | rs2328684 | T | G | 0.65 (0.61, 0.7) | 1.43E-33 | 0.24 | 0.25 |
| 20 | 21866898 | rs6047744 | T | G | 1.54 (1.43, 1.65) | 1.23E-33 | 0.24 | 0.25 |
| 20 | 21867054 | rs6047745 | C | A | 0.65 (0.61, 0.7) | 1.03E-33 | 0.24 | 0.25 |
| 20 | 21868688 | rs6113435 | G | C | 1.54 (1.44, 1.65) | 9.32E-34 | 0.24 | 0.25 |
| 20 | 21877052 | rs2424409 | G | C | 1.58 (1.41, 1.77) | 1.28E-14 | 0.63 | 0 |
| 20 | 21877056 | rs2424410 | C | A | 0.65 (0.61, 0.7) | 6.08E-34 | 0.25 | 0.23 |
| 20 | 21879684 | rs6035995 | G | A | 0.65 (0.61, 0.7) | 8.21E-34 | 0.26 | 0.23 |
| 20 | 21884484 | rs1080275 | G | A | 0.65 (0.61, 0.7) | 5.54E-34 | 0.25 | 0.23 |
| 20 | 21886282 | rs6047761 | C | A | 0.65 (0.61, 0.7) | 5.11E-34 | 0.25 | 0.23 |
| 20 | 21886530 | rs4815086 | C | A | 0.65 (0.61, 0.7) | 5.31E-34 | 0.26 | 0.23 |
| 20 | 21886604 | rs4815087 | G | C | 1.54 (1.44, 1.65) | 4.51E-34 | 0.25 | 0.23 |
| 20 | 21891804 | rs2424414 | T | C | 1.84 (1.51, 2.24) | 1.56E-09 | 0.68 | 0 |
| 20 | 21895184 | rs969921 | T | A | 0.65 (0.61, 0.69) | 3.96E-34 | 0.25 | 0.23 |
| 20 | 21897592 | rs6106434 | G | A | 1.38 (1.28, 1.49) | 1.82E-16 | 0.34 | 0.12 |
| 20 | 21899848 | rs2207878 | G | A | 0.66 (0.61, 0.71) | 2.99E-28 | 0.22 | 0.29 |
| 20 | 21900054 | rs970616 | C | A | 1.38 (1.28, 1.48) | 1.37E-16 | 0.5 | 0 |
| 20 | 21901648 | rs6047768 | G | A | 0.66 (0.61, 0.71) | 3.19E-28 | 0.24 | 0.26 |
| 20 | 21906048 | rs6047769 | G | A | 1.54 (1.44, 1.65) | 4.35E-34 | 0.24 | 0.25 |
| 20 | 21907906 | rs2424417 | T | A | 0.52 (0.43, 0.64) | 1.39E-09 | 0.73 | 0 |
| 20 | 21909410 | rs1555264 | G | A | 0.66 (0.61, 0.71) | 3.98E-28 | 0.31 | 0.17 |
| 20 | 21909472 | rs1555265 | T | C | 0.65 (0.61, 0.7) | 6.71E-34 | 0.25 | 0.23 |
| 20 | 21909964 | rs6036003 | G | A | 1.54 (1.44, 1.65) | 5.00E-34 | 0.25 | 0.24 |
| 20 | 21910332 | rs169311 | C | A | 1.54 (1.44, 1.66) | 2.37E-34 | 0.23 | 0.26 |
| 20 | 21911048 | rs2424420 | T | C | 1.58 (1.41, 1.78) | 7.59E-15 | 0.63 | 0 |
| 20 | 21915950 | rs201546 | G | A | 1.5 (1.31, 1.71) | 4.77E-09 | 0.89 | 0 |
| 20 | 21916220 | rs2424422 | C | A | 1.59 (1.41, 1.78) | 5.93E-15 | 0.62 | 0 |
| 20 | 21917512 | rs2424424 | T | C | 1.66 (1.42, 1.93) | 1.05E-10 | 0.28 | 0.19 |
| 20 | 21920580 | rs2009652 | T | C | 1.54 (1.44, 1.65) | 3.74E-34 | 0.24 | 0.25 |
| 20 | 21922590 | rs6036007 | T | C | 1.54 (1.44, 1.65) | 3.77E-34 | 0.24 | 0.25 |
| 20 | 21922836 | rs201548 | T | C | 1.54 (1.44, 1.65) | 3.77E-34 | 0.24 | 0.25 |
| 20 | 21923026 | rs6106438 | T | C | 1.54 (1.44, 1.66) | 3.13E-34 | 0.23 | 0.26 |
| 20 | 21925094 | rs6113456 | T | G | 1.55 (1.45, 1.66) | 8.92E-35 | 0.27 | 0.21 |
| 20 | 21933498 | rs6047798 | G | A | 1.54 (1.44, 1.65) | 2.82E-34 | 0.32 | 0.14 |
| 20 | 21933780 | rs1980551 | G | A | 0.65 (0.6, 0.69) | 1.66E-34 | 0.29 | 0.18 |
| 20 | 21934004 | rs1980552 | T | C | 0.65 (0.6, 0.69) | 1.53E-34 | 0.29 | 0.18 |
| 20 | 21934312 | rs6047799 | C | A | 1.61 (1.5, 1.72) | 1.36E-38 | 0.64 | 0 |
| 20 | 21935460 | rs6047801 | T | G | 1.61 (1.38, 1.88) | 1.46E-09 | 0.25 | 0.24 |
| 20 | 21936830 | rs127747 | G | A | 1.6 (1.49, 1.72) | 3.14E-39 | 0.56 | 0 |
| 20 | 21937972 | rs6047804 | T | C | 0.62 (0.53, 0.73) | 5.47E-09 | 0.16 | 0.37 |
| 20 | 21945116 | rs201559 | T | C | 0.63 (0.58, 0.67) | 3.92E-39 | 0.58 | 0 |
| 20 | 21947212 | rs201561 | G | C | 0.63 (0.58, 0.67) | 5.37E-39 | 0.59 | 0 |
| 20 | 21948174 | rs201562 | T | G | 1.5 (1.39, 1.61) | 1.13E-27 | 0.3 | 0.16 |
| 20 | 21948280 | rs201563 | T | C | 1.6 (1.49, 1.71) | 3.91E-39 | 0.56 | 0 |
| 20 | 21955602 | rs201564 | G | A | 1.47 (1.36, 1.58) | 9.74E-23 | 0.9 | 0 |
| 20 | 21957336 | rs201565 | T | C | 1.6 (1.49, 1.71) | 3.65E-39 | 0.55 | 0 |
| 20 | 21960862 | rs201569 | T | C | 1.46 (1.36, 1.58) | 1.01E-22 | 0.89 | 0 |
| 20 | 21961514 | rs201571 | T | C | 1.52 (1.41, 1.64) | 9.73E-28 | 0.79 | 0 |
| 20 | 21962754 | rs201572 | T | C | 1.52 (1.41, 1.64) | 7.83E-28 | 0.79 | 0 |
| 20 | 21963564 | rs201574 | T | C | 0.63 (0.58, 0.67) | 2.96E-39 | 0.55 | 0 |
| 20 | 21975674 | rs201587 | G | A | 0.62 (0.58, 0.67) | 2.44E-39 | 0.56 | 0 |
| 20 | 21975788 | rs4815089 | T | C | 0.62 (0.53, 0.73) | 5.64E-09 | 0.16 | 0.38 |
| 20 | 21981560 | rs6047837 | T | C | 1.59 (1.37, 1.85) | 1.58E-09 | 0.23 | 0.26 |
| 20 | 21981820 | rs201593 | G | A | 0.62 (0.58, 0.67) | 1.80E-39 | 0.57 | 0 |
| 20 | 21981920 | rs6047838 | G | A | 0.66 (0.58, 0.75) | 5.64E-10 | 0.25 | 0.25 |
| 20 | 21982672 | rs201594 | T | C | 1.6 (1.49, 1.72) | 2.29E-39 | 0.58 | 0 |
| 20 | 21983416 | rs4815090 | T | G | 0.67 (0.62, 0.73) | 4.43E-21 | 0.9 | 0 |
| 20 | 21984524 | rs6113483 | G | C | 1.47 (1.36, 1.58) | 4.72E-23 | 0.9 | 0 |
| **20** | **21985576** | **rs6047844** | **T** | **C** | **1.6 (1.49, 1.72)** | **1.71E-39** | **0.58** | **0** |
| 20 | 21986726 | rs6137526 | T | C | 0.68 (0.63, 0.74) | 6.63E-23 | 0.89 | 0 |
| 20 | 21988422 | rs6132477 | T | C | 0.68 (0.63, 0.74) | 4.98E-23 | 0.89 | 0 |
| 20 | 21988956 | rs16983348 | G | A | 0.62 (0.53, 0.73) | 3.39E-09 | 0.17 | 0.36 |
| 20 | 21990418 | rs913063 | C | A | 0.63 (0.59, 0.68) | 1.57E-38 | 0.26 | 0.22 |
| 20 | 21991920 | rs6137527 | T | C | 0.68 (0.63, 0.74) | 4.27E-23 | 0.89 | 0 |
| 20 | 21992204 | rs6036026 | T | C | 1.59 (1.48, 1.7) | 1.51E-38 | 0.26 | 0.22 |
| 20 | 21995176 | rs4813445 | G | A | 1.59 (1.48, 1.7) | 1.48E-38 | 0.26 | 0.22 |
| 20 | 21997176 | rs6036029 | T | C | 0.63 (0.59, 0.68) | 1.48E-38 | 0.26 | 0.22 |
| 20 | 21997760 | rs4813446 | T | C | 1.59 (1.48, 1.7) | 1.47E-38 | 0.26 | 0.22 |
| 20 | 21998504 | rs1160312 | G | A | 0.63 (0.59, 0.68) | 1.47E-38 | 0.26 | 0.22 |
| 20 | 21999812 | rs1887056 | C | A | 0.63 (0.59, 0.68) | 1.39E-38 | 0.26 | 0.22 |
| 20 | 22000060 | rs16983367 | T | G | 0.62 (0.53, 0.73) | 2.47E-09 | 0.18 | 0.35 |
| 20 | 22005176 | rs2104504 | T | C | 1.6 (1.38, 1.86) | 6.10E-10 | 0.26 | 0.22 |
| 20 | 22005416 | rs6113491 | C | A | 0.63 (0.59, 0.68) | 3.10E-37 | 0.31 | 0.16 |
| 20 | 22009488 | rs6113495 | G | A | 0.68 (0.63, 0.74) | 3.68E-23 | 0.89 | 0 |
| 20 | 22009632 | rs6113496 | T | G | 0.63 (0.59, 0.67) | 1.02E-38 | 0.26 | 0.23 |
| 20 | 22012972 | rs6113501 | G | A | 1.47 (1.36, 1.58) | 3.79E-23 | 0.89 | 0 |
| 20 | 22013466 | rs11698943 | G | A | 0.68 (0.63, 0.74) | 4.50E-23 | 0.9 | 0 |
| 20 | 22014992 | rs10485628 | G | A | 1.6 (1.38, 1.86) | 5.63E-10 | 0.26 | 0.22 |
| 20 | 22018524 | rs6036037 | C | A | 0.63 (0.59, 0.68) | 8.62E-37 | 0.26 | 0.23 |
| 20 | 22018664 | rs6137533 | T | C | 0.68 (0.63, 0.74) | 3.64E-23 | 0.89 | 0 |
| 20 | 22022096 | rs4813447 | G | A | 1.6 (1.38, 1.85) | 6.48E-10 | 0.26 | 0.23 |
| 20 | 22024188 | rs6113508 | G | A | 0.68 (0.63, 0.74) | 4.28E-23 | 0.89 | 0 |
| 20 | 22025352 | rs6113509 | G | A | 1.47 (1.36, 1.58) | 4.22E-23 | 0.89 | 0 |
| 20 | 22028540 | rs1883751 | G | A | 1.47 (1.36, 1.58) | 4.47E-23 | 0.89 | 0 |
| 20 | 22029332 | rs4815092 | G | C | 0.67 (0.62, 0.73) | 2.00E-22 | 0.91 | 0 |
| 20 | 22030448 | rs6036041 | G | A | 0.65 (0.6, 0.7) | 2.04E-28 | 0.45 | 0 |
| 20 | 22034416 | rs201141 | T | C | 0.62 (0.53, 0.72) | 2.24E-09 | 0.22 | 0.29 |
| 20 | 22034860 | rs201142 | G | A | 0.62 (0.53, 0.72) | 2.24E-09 | 0.22 | 0.29 |
| 20 | 22035538 | rs201144 | G | A | 1.6 (1.38, 1.86) | 9.48E-10 | 0.3 | 0.18 |
| 20 | 22035670 | rs201145 | G | A | 1.59 (1.48, 1.71) | 2.05E-38 | 0.3 | 0.17 |
| 20 | 22036650 | rs201146 | C | A | 0.63 (0.59, 0.67) | 1.93E-38 | 0.3 | 0.17 |
| 20 | 22037084 | rs201147 | T | A | 0.62 (0.53, 0.72) | 2.46E-09 | 0.26 | 0.24 |
| 20 | 22038388 | rs201149 | T | A | 0.63 (0.59, 0.68) | 5.02E-37 | 0.29 | 0.19 |
| 20 | 22038580 | rs201150 | T | A | 0.63 (0.59, 0.68) | 7.44E-37 | 0.3 | 0.17 |
| 20 | 22050152 | rs6047915 | T | C | 0.62 (0.53, 0.72) | 2.48E-09 | 0.26 | 0.23 |
| 20 | 22052420 | rs6047917 | G | A | 1.59 (1.48, 1.7) | 6.03E-37 | 0.29 | 0.18 |
| 20 | 22053844 | rs2206754 | T | C | 0.62 (0.53, 0.72) | 3.67E-09 | 0.21 | 0.29 |
| 20 | 22054230 | rs11698068 | G | A | 1.59 (1.48, 1.7) | 7.12E-37 | 0.29 | 0.19 |
| 20 | 22055728 | rs2328700 | T | C | 0.62 (0.53, 0.72) | 3.60E-09 | 0.21 | 0.29 |
| 20 | 22057896 | rs6137547 | G | A | 0.67 (0.62, 0.73) | 1.25E-22 | 0.78 | 0 |
| 20 | 22059034 | rs1303872 | T | C | 1.56 (1.45, 1.68) | 9.58E-33 | 0.42 | 0.01 |
| 20 | 22059380 | rs1303873 | T | C | 0.62 (0.53, 0.72) | 3.34E-09 | 0.22 | 0.29 |
| 20 | 22063728 | rs804516 | G | A | 1.58 (1.47, 1.7) | 2.38E-36 | 0.32 | 0.15 |
| 20 | 22065778 | rs6113534 | G | C | 0.68 (0.62, 0.73) | 4.09E-22 | 0.84 | 0 |
| 20 | 22067140 | rs804520 | G | A | 1.56 (1.46, 1.68) | 6.82E-35 | 0.36 | 0.08 |
| 20 | 22067264 | rs804521 | G | A | 1.61 (1.39, 1.88) | 1.05E-09 | 0.3 | 0.17 |
| 20 | 22067832 | rs973106 | G | A | 1.48 (1.37, 1.6) | 4.95E-22 | 0.84 | 0 |
| 20 | 22068288 | rs804522 | T | C | 1.61 (1.39, 1.88) | 1.04E-09 | 0.3 | 0.17 |
| 20 | 22070158 | rs708996 | G | C | 0.64 (0.6, 0.69) | 2.66E-32 | 0.41 | 0.01 |
| 20 | 22070810 | rs804524 | T | C | 0.64 (0.6, 0.69) | 7.66E-35 | 0.37 | 0.08 |
| 20 | 22071128 | rs6113539 | G | A | 1.48 (1.37, 1.6) | 4.11E-22 | 0.85 | 0 |
| 20 | 22071208 | rs804525 | C | A | 0.61 (0.52, 0.72) | 2.48E-09 | 0.22 | 0.28 |
| 20 | 22072416 | rs804527 | T | G | 1.62 (1.39, 1.88) | 1.01E-09 | 0.3 | 0.17 |
| 20 | 22073884 | rs804529 | C | A | 1.62 (1.39, 1.88) | 1.01E-09 | 0.3 | 0.17 |
| 20 | 22075048 | rs708997 | C | A | 1.62 (1.39, 1.88) | 1.01E-09 | 0.3 | 0.17 |
| 20 | 22075894 | rs708999 | T | C | 1.61 (1.39, 1.88) | 1.04E-09 | 0.3 | 0.17 |
| 20 | 22076470 | rs804530 | T | C | 0.61 (0.52, 0.72) | 2.58E-09 | 0.22 | 0.29 |
| 20 | 22076554 | rs804531 | G | A | 0.65 (0.61, 0.7) | 2.89E-29 | 0.54 | 0 |
| 20 | 22077324 | rs6113543 | G | A | 0.68 (0.63, 0.73) | 5.25E-22 | 0.86 | 0 |
| 20 | 22077556 | rs804532 | G | A | 0.61 (0.52, 0.72) | 2.72E-09 | 0.22 | 0.29 |
| 20 | 22077932 | rs804533 | G | A | 0.64 (0.6, 0.69) | 1.09E-34 | 0.38 | 0.06 |
| 20 | 22078528 | rs1415798 | G | C | 0.68 (0.63, 0.73) | 4.40E-22 | 0.86 | 0 |
| 20 | 22079194 | rs1415799 | T | C | 0.68 (0.62, 0.73) | 4.07E-22 | 0.75 | 0 |
| 20 | 22083308 | rs804536 | T | C | 0.62 (0.53, 0.73) | 5.83E-09 | 0.15 | 0.39 |
| 20 | 22084000 | rs2328701 | G | C | 0.64 (0.59, 0.69) | 1.51E-34 | 0.32 | 0.15 |
| 20 | 22084092 | rs804537 | T | C | 1.6 (1.37, 1.86) | 3.38E-09 | 0.22 | 0.27 |
| 20 | 22084272 | rs804538 | G | A | 0.62 (0.53, 0.73) | 1.12E-08 | 0.15 | 0.38 |
| 20 | 22084708 | rs804539 | G | C | 0.62 (0.53, 0.73) | 1.55E-08 | 0.15 | 0.38 |
| 20 | 22084786 | rs6047929 | G | A | 1.49 (1.38, 1.61) | 8.13E-25 | 0.79 | 0 |
| 20 | 22084840 | rs6113548 | T | C | 1.48 (1.37, 1.61) | 4.16E-22 | 0.53 | 0 |
| 20 | 22087448 | rs1090265 | T | C | 0.62 (0.53, 0.73) | 1.79E-08 | 0.16 | 0.38 |
| 20 | 22087934 | rs6113551 | T | A | 1.38 (1.28, 1.49) | 3.70E-16 | 0.28 | 0.2 |
| 20 | 22092268 | rs6137561 | G | A | 0.71 (0.66, 0.77) | 4.69E-19 | 0.43 | 0 |
| 20 | 22143064 | rs6113583 | T | C | 1.37 (1.27, 1.48) | 3.72E-15 | 0.17 | 0.36 |
| 20 | 22144024 | rs1159466 | G | C | 1.37 (1.27, 1.47) | 4.87E-17 | 0.55 | 0 |
| 20 | 22145212 | rs6137582 | G | A | 0.73 (0.68, 0.79) | 3.44E-16 | 0.13 | 0.39 |
| 20 | 22145686 | rs2876633 | T | A | 1.37 (1.27, 1.47) | 2.29E-16 | 0.14 | 0.38 |
| 20 | 22147504 | rs742655 | T | C | 0.74 (0.69, 0.8) | 3.89E-17 | 0.13 | 0.39 |
| 20 | 22148414 | rs804614 | G | A | 1.35 (1.26, 1.44) | 4.29E-17 | 0.13 | 0.39 |
| 20 | 22149176 | rs804615 | T | C | 0.74 (0.69, 0.8) | 2.82E-17 | 0.13 | 0.39 |
| 20 | 22149840 | rs804616 | T | C | 1.35 (1.26, 1.44) | 4.16E-17 | 0.13 | 0.39 |
| 20 | 22151480 | rs804617 | T | G | 1.35 (1.26, 1.44) | 3.61E-17 | 0.13 | 0.39 |
| 20 | 22151740 | rs709006 | T | C | 0.74 (0.69, 0.8) | 3.60E-17 | 0.13 | 0.39 |
| 20 | 22151788 | rs709007 | G | A | 1.35 (1.26, 1.44) | 3.54E-17 | 0.13 | 0.39 |
| 20 | 22152464 | rs804618 | G | A | 0.74 (0.69, 0.8) | 3.60E-17 | 0.13 | 0.39 |
| 20 | 22154968 | rs1090292 | T | C | 1.34 (1.26, 1.44) | 4.73E-17 | 0.12 | 0.41 |
| 20 | 22155156 | rs1090293 | G | A | 0.74 (0.69, 0.8) | 4.92E-17 | 0.12 | 0.4 |
| 20 | 22157538 | rs4815098 | G | A | 1.37 (1.27, 1.47) | 3.23E-16 | 0.14 | 0.38 |
| 20 | 22159414 | rs1415801 | T | G | 1.37 (1.27, 1.47) | 3.16E-16 | 0.14 | 0.38 |
| 20 | 22160946 | rs12625314 | T | C | 0.73 (0.68, 0.79) | 3.14E-16 | 0.14 | 0.38 |
| 20 | 22160962 | rs12625315 | T | C | 0.74 (0.69, 0.8) | 7.69E-14 | 0.18 | 0.35 |
| 20 | 22171640 | rs6106481 | G | A | 0.77 (0.71, 0.83) | 2.89E-11 | 0.35 | 0.1 |
| 20 | 22177504 | rs6113592 | G | A | 0.8 (0.74, 0.86) | 2.85E-09 | 0.36 | 0.09 |
| 20 | 22187448 | rs6047978 | C | A | 0.8 (0.74, 0.86) | 2.96E-09 | 0.36 | 0.08 |
| 20 | 22189466 | rs1337908 | T | A | 1.3 (1.2, 1.4) | 3.06E-11 | 0.35 | 0.11 |
| 20 | 22193868 | rs10485630 | T | G | 1.26 (1.17, 1.36) | 1.40E-09 | 0.41 | 0 |
| 20 | 22194450 | rs6047982 | T | G | 1.26 (1.17, 1.36) | 1.25E-09 | 0.43 | 0 |
| 20 | 22200276 | rs6113604 | T | C | 0.77 (0.72, 0.83) | 7.35E-11 | 0.39 | 0.03 |
| 20 | 22203000 | rs4815101 | G | C | 0.78 (0.72, 0.84) | 1.14E-10 | 0.34 | 0.12 |
| 20 | 22203172 | rs4815102 | T | G | 0.77 (0.72, 0.83) | 7.49E-11 | 0.4 | 0.03 |
| 20 | 22204270 | rs6113607 | T | C | 1.29 (1.19, 1.39) | 1.14E-10 | 0.34 | 0.12 |
| 20 | 22208552 | rs200366 | G | A | 1.35 (1.25, 1.45) | 1.97E-15 | 0.38 | 0.06 |
| 20 | 22208892 | rs6106493 | T | C | 0.77 (0.72, 0.84) | 7.85E-11 | 0.4 | 0.03 |
| 20 | 22210264 | rs1569781 | T | G | 0.77 (0.72, 0.84) | 7.93E-11 | 0.4 | 0.03 |
| 20 | 22210428 | rs1569782 | G | C | 0.78 (0.72, 0.84) | 1.20E-10 | 0.34 | 0.12 |
| 20 | 22211132 | rs2295099 | G | A | 1.31 (1.22, 1.41) | 3.16E-12 | 0.21 | 0.29 |
| 20 | 22213098 | rs200369 | C | A | 1.29 (1.2, 1.39) | 1.45E-11 | 0.19 | 0.33 |
| 20 | 22215058 | rs1028444 | T | C | 1.25 (1.16, 1.34) | 2.46E-09 | 0.5 | 0 |
| 20 | 22216484 | rs6047999 | T | C | 1.25 (1.16, 1.34) | 2.08E-09 | 0.51 | 0 |
| 20 | 22219088 | rs1832371 | G | A | 1.25 (1.16, 1.34) | 2.22E-09 | 0.51 | 0 |
| 20 | 22222860 | rs183216 | T | A | 0.77 (0.72, 0.83) | 1.20E-11 | 0.22 | 0.29 |
| 20 | 22222930 | rs980970 | T | C | 0.8 (0.74, 0.86) | 2.38E-09 | 0.51 | 0 |
| 20 | 22223648 | rs199780 | T | G | 0.77 (0.72, 0.83) | 1.20E-11 | 0.22 | 0.29 |
| 20 | 22224484 | rs199781 | G | C | 0.78 (0.72, 0.83) | 1.80E-11 | 0.16 | 0.37 |
| 20 | 22224812 | rs4813452 | T | G | 1.25 (1.16, 1.34) | 3.67E-09 | 0.48 | 0 |
| 20 | 22228604 | rs6113622 | T | C | 1.25 (1.16, 1.34) | 2.76E-09 | 0.51 | 0 |
| 20 | 22229872 | rs17192817 | T | C | 1.24 (1.16, 1.34) | 4.03E-09 | 0.49 | 0 |
| 20 | 22230356 | rs172036 | T | C | 0.77 (0.72, 0.83) | 9.43E-12 | 0.2 | 0.31 |
| 20 | 22231878 | rs199790 | T | C | 1.29 (1.2, 1.39) | 1.32E-11 | 0.17 | 0.35 |
| 20 | 22235304 | rs199793 | G | A | 0.78 (0.72, 0.83) | 7.49E-13 | 0.32 | 0.14 |
| 20 | 22238780 | rs8124887 | G | A | 1.23 (1.15, 1.33) | 2.57E-08 | 0.54 | 0 |
| 20 | 22239220 | rs4815103 | C | A | 1.23 (1.15, 1.33) | 2.40E-08 | 0.53 | 0 |
| 20 | 22239932 | rs199796 | T | C | 1.28 (1.19, 1.37) | 7.59E-11 | 0.2 | 0.3 |
| 20 | 22248304 | rs169230 | G | A | 1.25 (1.16, 1.35) | 4.30E-09 | 0.13 | 0.4 |
| X | 65309361 | rs7054364 | A | C | 0.71 (0.67,0.76) | 1.33E-25 | 0.77 | 0 |
| X | 65325170 | rs6624875 | A | G | 0.71 (0.66,0.75) | 2.90E-27 | 0.73 | 0 |
| X | 65331899 | rs2206203 | C | A | 0.71 (0.66,0.75) | 1.77E-27 | 0.66 | 0 |
| X | 65332812 | rs1011526 | A | G | 0.71 (0.66,0.75) | 1.77E-27 | 0.66 | 0 |
| X | 65343765 | rs806607 | C | T | 0.70 (0.65,0.74) | 3.11E-29 | 0.56 | 0 |
| X | 65345254 | rs806608 | T | G | 0.70 (0.66,0.74) | 5.05E-29 | 0.67 | 0 |
| X | 65350264 | rs806610 | C | T | 0.70 (0.65,0.74) | 4.82E-29 | 0.65 | 0 |
| X | 65351982 | rs1264216 | G | T | 0.70 (0.65,0.74) | 4.82E-29 | 0.65 | 0 |
| X | 65356189 | rs1091486 | T | C | 0.70 (0.66,0.74) | 5.05E-29 | 0.67 | 0 |
| X | 65433403 | rs5919042 | G | A | 0.70 (0.65,0.75) | 1.84E-26 | 0.71 | 0 |
| X | 65433624 | rs5919043 | C | T | 0.70 (0.65,0.75) | 1.84E-26 | 0.71 | 0 |
| X | 65448584 | rs601552 | A | G | 0.59 (0.54,0.65) | 1.01E-29 | 0.48 | 0 |
| X | 65448903 | rs1463435 | C | T | 0.78 (0.73,0.83) | 5.08E-16 | 0.55 | 0 |
| X | 65455348 | rs6624177 | C | T | 0.67 (0.63,0.72) | 8.23E-31 | 0.59 | 0 |
| X | 65491314 | rs1585131 | C | A | 0.67 (0.63,0.72) | 2.15E-30 | 0.59 | 0 |
| X | 65521580 | rs5964522 | T | A | 0.66 (0.61,0.70) | 1.24E-34 | 0.66 | 0 |
| X | 65523086 | rs1379146 | T | A | 0.66 (0.61,0.70) | 1.32E-34 | 0.66 | 0 |
| X | 65523281 | rs2840240 | C | G | 0.66 (0.61,0.70) | 1.24E-34 | 0.66 | 0 |
| X | 65528029 | rs1926341 | C | T | 0.66 (0.61,0.70) | 7.12E-35 | 0.66 | 0 |
| X | 65544962 | rs5965189 | G | A | 0.66 (0.61,0.70) | 8.97E-35 | 0.81 | 0 |
| X | 65545588 | rs4240047 | A | T | 0.66 (0.61,0.70) | 8.97E-35 | 0.81 | 0 |
| X | 65546997 | rs7888975 | G | A | 0.65 (0.61,0.70) | 6.94E-35 | 0.77 | 0 |
| X | 65549504 | rs5965192 | A | G | 0.66 (0.61,0.70) | 8.15E-35 | 0.79 | 0 |
| X | 65574614 | rs4827479 | T | C | 0.74 (0.68,0.80) | 9.24E-13 | 0.38 | 0.05 |
| X | 65578623 | rs5919100 | T | G | 0.62 (0.58,0.66) | 4.36E-46 | 0.69 | 0 |
| X | 65586966 | rs4361890 | C | T | 0.62 (0.58,0.66) | 2.28E-46 | 0.69 | 0 |
| X | 65589063 | rs4240049 | C | T | 0.62 (0.58,0.66) | 2.28E-46 | 0.69 | 0 |
| X | 65589137 | rs5919108 | T | C | 0.62 (0.58,0.66) | 1.83E-46 | 0.67 | 0 |
| X | 65589319 | rs4357442 | A | G | 0.62 (0.58,0.66) | 1.83E-46 | 0.67 | 0 |
| X | 65589526 | rs5919110 | A | T | 0.71 (0.65,0.77) | 1.14E-16 | 0.73 | 0 |
| X | 65635063 | rs5919135 | T | C | 0.62 (0.58,0.66) | 5.03E-47 | 0.70 | 0 |
| X | 65640583 | rs1331101 | A | C | 0.62 (0.58,0.66) | 9.09E-47 | 0.74 | 0 |
| X | 65650569 | rs5918648 | C | T | 0.57 (0.52,0.63) | 6.21E-31 | 0.88 | 0 |
| X | 65725320 | rs1586315 | T | C | 0.61 (0.57,0.65) | 1.51E-49 | 0.56 | 0 |
| X | 65730685 | rs4827379 | T | C | 0.71 (0.65,0.77) | 1.14E-16 | 0.73 | 0 |
| X | 65741711 | rs1385699 | C | T | 0.59 (0.55,0.63) | 3.00E-53 | 0.42 | 0 |
| X | 65760568 | rs1352015 | C | T | 0.59 (0.55,0.63) | 3.00E-53 | 0.42 | 0 |
| X | 65831379 | rs5919174 | G | A | 0.46 (0.42,0.50) | 7.92E-76 | 0.83 | 0 |
| X | 65838758 | rs5919175 | G | A | 0.46 (0.42,0.50) | 5.23E-76 | 0.83 | 0 |
| X | 65849574 | rs775358 | T | C | 0.46 (0.42,0.49) | 2.33E-81 | 0.53 | 0 |
| X | 65869018 | rs16990143 | G | A | 0.46 (0.42,0.49) | 7.30E-78 | 0.83 | 0 |
| X | 65895898 | rs775362 | T | C | 0.46 (0.42,0.49) | 7.30E-78 | 0.83 | 0 |
| X | 65933752 | rs3843789 | T | C | 0.48 (0.44,0.54) | 5.42E-42 | 0.98 | 0 |
| X | 65964070 | rs5919200 | G | A | 0.45 (0.42,0.49) | 6.37E-83 | 0.77 | 0 |
| X | 65985316 | rs4827384 | G | A | 0.49 (0.44,0.54) | 7.61E-40 | 0.97 | 0 |
| X | 66002681 | rs1041668 | G | A | 0.45 (0.41,0.49) | 1.70E-83 | 0.62 | 0 |
| X | 66019047 | rs7878229 | C | T | 0.47 (0.42,0.53) | 3.24E-36 | 0.38 | 0.06 |
| X | 66031848 | rs4548330 | T | G | 0.44 (0.41,0.48) | 2.57E-86 | 0.66 | 0 |
| X | 66087225 | rs5919235 | G | A | 0.43 (0.40,0.47) | 4.62E-88 | 0.73 | 0 |
| X | 66138622 | rs7057795 | C | T | 0.44 (0.40,0.47) | 2.70E-83 | 0.75 | 0 |
| X | 66155042 | rs471205 | T | C | 0.67 (0.64,0.72) | 5.11E-39 | 0.64 | 0 |
| X | 66155654 | rs476709 | T | C | 0.44 (0.40,0.47) | 2.23E-83 | 0.74 | 0 |
| X | 66175639 | rs505520 | C | A | 0.42 (0.38,0.46) | 4.68E-84 | 0.72 | 0 |
| X | 66179398 | rs574001 | C | A | 0.42 (0.39,0.46) | 1.30E-83 | 0.59 | 0 |
| X | 66183973 | rs532649 | G | A | 0.42 (0.39,0.46) | 7.13E-87 | 0.75 | 0 |
| X | 66184927 | rs485454 | G | A | 0.42 (0.38,0.46) | 1.22E-83 | 0.70 | 0 |
| X | 66185099 | rs489099 | C | T | 0.42 (0.39,0.46) | 9.94E-87 | 0.75 | 0 |
| X | 66190296 | rs531840 | T | C | 0.42 (0.39,0.46) | 4.26E-86 | 0.76 | 0 |
| X | 66204409 | rs5919247 | C | T | 0.42 (0.39,0.46) | 7.68E-87 | 0.76 | 0 |
| X | 66204780 | rs5918688 | A | G | 0.42 (0.38,0.46) | 2.81E-76 | 0.58 | 0 |
| X | 66214912 | rs4827524 | A | G | 0.42 (0.39,0.46) | 6.45E-87 | 0.76 | 0 |
| X | 66217759 | rs989345 | G | A | 0.42 (0.39,0.46) | 2.74E-87 | 0.75 | 0 |
| X | 66221281 | rs5919266 | G | T | 0.42 (0.39,0.46) | 3.39E-86 | 0.70 | 0 |
| X | 66228526 | rs1567524 | T | C | 0.42 (0.39,0.46) | 5.93E-87 | 0.76 | 0 |
| X | 66232455 | rs5919270 | G | A | 0.42 (0.39,0.46) | 5.09E-87 | 0.76 | 0 |
| X | 66232849 | rs1511061 | C | T | 0.42 (0.38,0.45) | 6.82E-90 | 0.71 | 0 |
| X | 66233156 | rs1511060 | C | T | 0.42 (0.39,0.46) | 1.62E-86 | 0.77 | 0 |
| X | 66235493 | rs5918694 | T | C | 0.42 (0.39,0.46) | 1.01E-86 | 0.76 | 0 |
| X | 66238687 | rs4827392 | C | A | 0.42 (0.39,0.46) | 5.77E-87 | 0.76 | 0 |
| X | 66242222 | rs5919272 | G | A | 0.42 (0.39,0.46) | 5.10E-87 | 0.76 | 0 |
| X | 66242280 | rs5918696 | T | C | 0.42 (0.39,0.46) | 3.73E-87 | 0.74 | 0 |
| X | 66246877 | rs4827527 | G | A | 0.42 (0.39,0.46) | 7.97E-87 | 0.73 | 0 |
| X | 66253452 | rs938059 | C | A | 0.42 (0.39,0.46) | 9.42E-87 | 0.75 | 0 |
| X | 66253825 | rs938058 | G | A | 0.42 (0.39,0.46) | 9.42E-87 | 0.75 | 0 |
| X | 66254894 | rs981065 | C | T | 0.42 (0.39,0.46) | 9.07E-87 | 0.76 | 0 |
| X | 66264409 | rs1988995 | T | C | 0.42 (0.39,0.46) | 9.91E-86 | 0.74 | 0 |
| X | 66265361 | rs1511058 | C | T | 0.42 (0.39,0.46) | 7.47E-86 | 0.76 | 0 |
| X | 66269421 | rs1397631 | T | C | 0.47 (0.41,0.52) | 3.37E-36 | 0.48 | 0 |
| X | 66269660 | rs984094 | A | G | 0.42 (0.39,0.46) | 1.17E-86 | 0.74 | 0 |
| X | 66275691 | rs5964588 | T | G | 0.42 (0.39,0.46) | 1.22E-86 | 0.75 | 0 |
| X | 66275897 | rs5919285 | G | A | 0.42 (0.39,0.46) | 1.22E-86 | 0.75 | 0 |
| X | 66277339 | rs5919287 | T | C | 0.42 (0.39,0.46) | 1.22E-86 | 0.75 | 0 |
| X | 66291862 | rs1027970 | C | T | 0.42 (0.39,0.46) | 1.24E-86 | 0.76 | 0 |
| X | 66293341 | rs2221799 | G | A | 0.42 (0.39,0.46) | 1.67E-86 | 0.76 | 0 |
| X | 66304361 | rs4272525 | T | C | 0.42 (0.39,0.46) | 2.82E-86 | 0.72 | 0 |
| X | 66311578 | rs5919309 | T | C | 0.42 (0.39,0.46) | 4.62E-86 | 0.72 | 0 |
| X | 66322154 | rs12007727 | A | G | 0.43 (0.38,0.48) | 2.60E-49 | 0.96 | 0 |
| X | 66329586 | rs5918719 | C | T | 0.42 (0.39,0.46) | 1.75E-86 | 0.73 | 0 |
| X | 66336675 | rs5919321 | G | A | 0.42 (0.39,0.46) | 3.89E-86 | 0.72 | 0 |
| X | 66347396 | rs5919325 | A | G | 0.42 (0.39,0.46) | 2.97E-86 | 0.73 | 0 |
| X | 66351646 | rs2335503 | C | T | 0.42 (0.39,0.46) | 3.27E-86 | 0.72 | 0 |
| X | 66360222 | rs5919335 | A | G | 0.46 (0.42,0.50) | 1.62E-83 | 0.37 | 0.07 |
| X | 66365693 | rs6625150 | A | C | 0.46 (0.42,0.50) | 2.73E-83 | 0.34 | 0.12 |
| X | 66366212 | rs12009759 | A | G | 0.49 (0.44,0.54) | 1.35E-46 | 0.44 | 0 |
| X | 66393594 | rs2335868 | T | C | 0.46 (0.42,0.50) | 2.80E-83 | 0.35 | 0.10 |
| X | 66398525 | rs12558842 | C | A | 0.46 (0.42,0.49) | 4.86E-85 | 0.38 | 0.05 |
| X | 66398618 | rs6625155 | C | T | 0.46 (0.42,0.49) | 4.16E-85 | 0.38 | 0.07 |
| X | 66414536 | rs5918737 | T | C | 0.46 (0.42,0.50) | 5.52E-83 | 0.34 | 0.12 |
| X | 66428828 | rs5919363 | G | A | 0.46 (0.43,0.50) | 9.49E-86 | 0.11 | 0.44 |
| X | 66436233 | rs5965383 | G | T | 0.46 (0.43,0.50) | 2.14E-85 | 0.12 | 0.43 |
| X | 66436439 | rs2335506 | G | A | 0.46 (0.43,0.50) | 4.71E-85 | 0.13 | 0.41 |
| X | 66437375 | rs2335508 | A | G | 0.46 (0.43,0.50) | 4.70E-85 | 0.12 | 0.43 |
| X | 66447257 | rs6625174 | G | A | 0.46 (0.43,0.50) | 1.30E-85 | 0.13 | 0.41 |
| X | 66455454 | rs2878642 | A | G | 0.46 (0.43,0.50) | 1.63E-84 | 0.13 | 0.41 |
| X | 66456836 | rs2336175 | A | G | 0.46 (0.43,0.50) | 2.26E-80 | 0.13 | 0.42 |
| X | 66457823 | rs4484837 | G | A | 0.48 (0.42,0.54) | 6.69E-34 | 0.14 | 0.39 |
| X | 66471565 | rs5918745 | T | C | 0.45 (0.42,0.49) | 3.21E-91 | 0.21 | 0.30 |
| X | 66475061 | rs2497936 | T | C | 0.48 (0.44,0.52) | 1.64E-67 | 0.60 | 0 |
| **X** | **66479743** | **rs2497938** | **C** | **T** | **0.45 (0.42,0.49)** | **2.40E-91** | **0.19** | **0.32** |
| X | 66480739 | rs2497939 | C | A | 0.46 (0.42,0.50) | 4.01E-82 | 0.11 | 0.45 |
| X | 66481077 | rs2223842 | C | A | 0.46 (0.43,0.50) | 2.42E-85 | 0.10 | 0.45 |
| X | 66482821 | rs2497943 | G | T | 0.46 (0.43,0.50) | 2.51E-84 | 0.10 | 0.46 |
| X | 66483175 | rs2473897 | C | T | 0.46 (0.43,0.50) | 8.34E-85 | 0.10 | 0.46 |
| X | 66486896 | rs2223841 | C | T | 0.46 (0.43,0.50) | 3.90E-85 | 0.10 | 0.45 |
| X | 66487452 | rs2473896 | C | T | 0.46 (0.43,0.50) | 5.01E-85 | 0.10 | 0.45 |
| X | 66487762 | rs2473895 | T | C | 0.46 (0.43,0.50) | 6.85E-85 | 0.10 | 0.45 |
| X | 66490468 | rs2207080 | G | A | 0.46 (0.43,0.50) | 1.47E-84 | 0.10 | 0.45 |
| X | 66493049 | rs721451 | G | A | 0.46 (0.43,0.50) | 7.80E-83 | 0.12 | 0.43 |
| X | 66493198 | rs2473891 | T | C | 0.46 (0.43,0.50) | 5.19E-86 | 0.11 | 0.44 |
| X | 66529906 | rs2473870 | G | A | 0.46 (0.43,0.50) | 2.19E-83 | 0.11 | 0.45 |
| X | 66544165 | rs2497911 | A | C | 0.46 (0.43,0.50) | 2.23E-83 | 0.10 | 0.45 |
| X | 66549068 | rs2497917 | A | G | 0.46 (0.43,0.50) | 1.83E-83 | 0.10 | 0.45 |
| X | 66564941 | rs2497928 | C | A | 0.46 (0.43,0.50) | 1.66E-85 | 0.13 | 0.42 |
| X | 66569429 | rs2497930 | C | A | 0.46 (0.43,0.50) | 2.26E-85 | 0.12 | 0.43 |
| X | 66580991 | rs2497935 | G | A | 0.46 (0.43,0.50) | 2.26E-85 | 0.12 | 0.43 |
| X | 66624114 | rs2207041 | A | C | 0.50 (0.46,0.54) | 4.04E-68 | 0.23 | 0.27 |
| X | 66662689 | rs962458 | G | A | 0.46 (0.41,0.52) | 1.66E-38 | 0.36 | 0.08 |
| X | 66668280 | rs7888856 | G | A | 0.50 (0.46,0.54) | 1.95E-68 | 0.32 | 0.15 |
| X | 66693791 | rs12396249 | A | G | 0.49 (0.46,0.54) | 9.90E-69 | 0.34 | 0.12 |
| X | 66704950 | rs1204038 | A | G | 0.49 (0.45,0.53) | 6.32E-70 | 0.37 | 0.07 |
| X | 66714836 | rs2255702 | T | C | 0.49 (0.45,0.53) | 6.32E-70 | 0.37 | 0.07 |
| X | 66722755 | rs5918757 | G | A | 0.49 (0.45,0.53) | 5.50E-70 | 0.36 | 0.09 |
| X | 66742082 | rs5919393 | C | T | 0.50 (0.46,0.54) | 1.85E-68 | 0.35 | 0.10 |
| X | 66745110 | rs4827545 | A | G | 0.50 (0.46,0.54) | 1.55E-68 | 0.33 | 0.13 |
| X | 66755476 | rs5918760 | T | C | 0.49 (0.46,0.54) | 8.63E-69 | 0.38 | 0.06 |
| X | 66792481 | rs6624304 | T | C | 0.51 (0.47,0.55) | 8.53E-62 | 0.36 | 0.08 |
| X | 66795644 | rs1337080 | G | A | 0.50 (0.45,0.56) | 2.91E-35 | 0.49 | 0 |
| X | 66831526 | rs5918762 | T | C | 0.51 (0.47,0.55) | 8.53E-62 | 0.36 | 0.08 |
| X | 66854432 | rs5918764 | T | C | 0.50 (0.45,0.56) | 2.91E-35 | 0.49 | 0 |
| X | 66873156 | rs5964607 | T | C | 0.65 (0.61,0.69) | 1.09E-37 | 0.45 | 0 |
| X | 66876367 | rs1931542 | A | G | 0.65 (0.61,0.69) | 1.03E-37 | 0.45 | 0 |
| X | 66881843 | rs1572502 | C | T | 0.65 (0.61,0.69) | 1.02E-37 | 0.45 | 0 |
| X | 66884958 | rs1415271 | G | T | 0.65 (0.61,0.69) | 1.00E-37 | 0.43 | 0 |
| X | 66885326 | rs5019585 | A | G | 0.65 (0.61,0.69) | 1.25E-37 | 0.46 | 0 |
| X | 66895296 | rs1931545 | G | T | 0.65 (0.61,0.69) | 1.09E-37 | 0.45 | 0 |
| X | 66898321 | rs3927643 | T | C | 0.65 (0.61,0.69) | 1.09E-37 | 0.45 | 0 |
| X | 66900243 | rs6625208 | C | T | 0.65 (0.61,0.69) | 1.20E-37 | 0.46 | 0 |
| X | 66904482 | rs9699051 | T | C | 0.50 (0.45,0.56) | 2.65E-33 | 0.71 | 0 |
| X | 66908084 | rs7057791 | T | C | 0.65 (0.61,0.69) | 1.44E-37 | 0.48 | 0 |
| X | 66913866 | rs12010636 | T | C | 0.50 (0.45,0.56) | 2.65E-33 | 0.71 | 0 |
| X | 66920309 | rs5919427 | G | T | 0.65 (0.61,0.69) | 1.15E-37 | 0.45 | 0 |
| X | 66934751 | rs4370673 | T | G | 0.66 (0.61,0.70) | 1.02E-35 | 0.44 | 0 |
| X | 66938275 | rs5919432 | C | T | 0.66 (0.61,0.70) | 7.27E-36 | 0.43 | 0 |
| X | 66944947 | rs4456006 | C | A | 0.65 (0.61,0.69) | 2.58E-38 | 0.34 | 0.12 |
| X | 66950662 | rs5964614 | T | G | 0.65 (0.61,0.70) | 8.33E-38 | 0.38 | 0.05 |
| X | 66979215 | rs4827556 | C | T | 0.65 (0.61,0.70) | 3.49E-37 | 0.41 | 0.02 |
| X | 67005788 | rs7885198 | A | G | 0.67 (0.62,0.71) | 1.15E-31 | 0.83 | 0 |
| X | 67018756 | rs2781516 | A | G | 0.68 (0.63,0.73) | 4.66E-23 | 0.59 | 0 |
| X | 67086147 | rs5965478 | T | G | 0.73 (0.69,0.78) | 1.44E-27 | 0.11 | 0.45 |
| X | 67088023 | rs2363785 | T | G | 0.73 (0.69,0.78) | 1.44E-27 | 0.11 | 0.45 |
| X | 67090057 | rs7050236 | A | G | 0.71 (0.66,0.75) | 1.03E-28 | 0.88 | 0 |
| X | 67090456 | rs16989069 | T | C | 0.71 (0.67,0.75) | 2.41E-28 | 0.87 | 0 |
| X | 67090515 | rs16990427 | A | G | 0.71 (0.66,0.75) | 1.41E-28 | 0.88 | 0 |
| X | 67093330 | rs5964632 | C | T | 0.73 (0.69,0.77) | 3.77E-28 | 0.11 | 0.45 |
| X | 67094419 | rs16990434 | T | C | 0.70 (0.66,0.75) | 4.57E-29 | 0.88 | 0 |
| X | 67094485 | rs4562482 | C | A | 0.73 (0.69,0.77) | 3.29E-28 | 0.11 | 0.45 |
| X | 67094528 | rs3898332 | G | A | 0.70 (0.66,0.75) | 5.19E-30 | 0.82 | 0 |
| X | 67100309 | rs5919492 | G | T | 0.71 (0.67,0.75) | 1.18E-29 | 0.79 | 0 |
| X | 67105253 | rs11094062 | G | A | 0.73 (0.69,0.78) | 6.49E-28 | 0.10 | 0.45 |
| X | 67119659 | rs12009526 | T | C | 0.70 (0.66,0.75) | 2.20E-30 | 0.77 | 0 |
| X | 67125660 | rs5918801 | T | G | 0.70 (0.66,0.75) | 2.20E-30 | 0.77 | 0 |
| X | 67168138 | rs12008699 | A | G | 0.69 (0.65,0.73) | 4.00E-33 | 0.79 | 0 |
| X | 67176649 | rs2765950 | C | T | 0.78 (0.74,0.83) | 3.69E-18 | 0.14 | 0.40 |
| X | 67202792 | rs16990499 | A | C | 0.69 (0.65,0.73) | 4.00E-33 | 0.79 | 0 |
| X | 67204578 | rs5918809 | G | A | 0.71 (0.67,0.75) | 1.12E-29 | 0.65 | 0 |
| X | 67210058 | rs17217221 | A | G | 0.70 (0.66,0.74) | 7.21E-30 | 0.53 | 0 |
| X | 67212853 | rs12013576 | C | T | 0.71 (0.67,0.75) | 2.17E-30 | 0.62 | 0 |
| X | 67281028 | rs7881511 | A | G | 1.27 (1.17,1.38) | 1.25E-08 | 0.15 | 0.39 |
| X | 67288202 | rs12011480 | T | C | 0.72 (0.68,0.76) | 3.26E-28 | 0.45 | 0 |
| X | 67298626 | rs12389669 | T | G | 1.27 (1.17,1.38) | 1.25E-08 | 0.15 | 0.39 |
| X | 67302669 | rs12854385 | C | A | 1.29 (1.18,1.40) | 3.20E-09 | 0.15 | 0.39 |

Abbreviation: Chr., chromosome.

Note: the lead SNPs at the genome-wide significant loci are in bold
