# Supplementary material for: Six Novel Susceptibility Loci for Early-Onset Androgenetic Alopecia and Their Unexpected Association with Common Diseases
Source: PLoS Genet. 2012 May 31;8(5):e1002746. doi: 10.1371/journal.pgen.1002746 (PMC3364959; doi:10.1371/journal.pgen.1002746)
Supplement: Table S3 — Average Expression Signal for All Genes Shown in Figure 1 across Three Tissue Types and Blood. (DOC) [file pgen.1002746.s006.doc]

**Table S3 Average Expression Signal for All Genes Shown in Figure 1 across Three Tissue Types and Blood**

| **GENE** | **Chr.** | **Illumina PROBE_ID** | **Hair** | | **Skin** | | **Scalp (temple)** | | **Blood** | |
| --- | --- | --- | --- | --- | --- | --- | --- | --- | --- | --- |
|  |  |  | **AVG Signal** | **Detection p value** | **AVG Signal** | **Detection p value** | **AVG Signal** | **Detection p value** | **AVG Signal** | **Detection p value** |
| ***ANGPTL7*** | 1 | ILMN_1813361 | 562.8 | < 10E-03 | 16.2 | 9.22E-03 | 236.9 | < 10E-03 | - | not significant |
| ***C1orf127*** | 1 | ILMN_1744089 | 30.3 | 9.22E-03 | 22.5 | 2.64E-03 | 10.6 | 4.87E-02 | 29.8 | not significant |
| ***CASZ1*** | 1 | ILMN_1655191 | 285.8 | 1.32E-03 | 287.3 | < 10E-03 | 410.6 | < 10E-03 | 122.7 | 2.64E-03 |
| ***EXOSC10*** | 1 | ILMN_1670796 | 245.0 | 1.32E-03 | 299.6 | < 10E-03 | 346.4 | < 10E-03 | 182.1 | < 10E-03 |
| ***MASP2*** | 1 | ILMN_1709408 | - | not significant | - | not significant | - | not significant | - | not significant |
| ***MTOR*** | 1 | ILMN_1769031 | 90.7 | 2.64E-03 | 277.1 | < 10E-03 | 286.4 | < 10E-03 | 115.0 | 3.95E-03 |
| ***PEX14*** | 1 | ILMN_1763634 | 60.9 | 2.64E-03 | 33.7 | < 10E-03 | 44.6 | < 10E-03 | 28.9 | not significant |
| ***SRM*** | 1 | ILMN_1661337 | 180.0 | 2.64E-03 | 84.4 | < 10E-03 | 79.0 | < 10E-03 | 220.4 | < 10E-03 |
| ***TARDBP*** | 1 | ILMN_1677532 | 4.4 | not significant | 44.8 | < 10E-03 | 54.9 | < 10E-03 | 70.8 | 1.32E-02 |
| ***UBIAD1*** | 1 | ILMN_1651872 | 36.9 | 6.59E-03 | 632.1 | < 10E-03 | 390.6 | < 10E-03 | 24.8 | not significant |
| ***ASB1*** | 2 | ILMN_1683096 | 26.0 | 1.32E-02 | 45.8 | < 10E-03 | 60.7 | < 10E-03 | 2.3 | not significant |
| ***ESPNL*** | 2 | ILMN_2115862 | - | not significant | - | not significant | - | not significant | - | not significant |
| ***HDAC4*** | 2 | ILMN_1764396 | 84.1 | 2.64E-03 | 63.5 | < 10E-03 | 102.2 | < 10E-03 | 79.2 | 1.05E-02 |
| ***HES6*** | 2 | ILMN_1694268 | 82.4 | 2.64E-03 | 19.0 | 6.59E-03 | 44.3 | < 10E-03 | 74.2 | 1.19E-02 |
| ***KLHL30*** | 2 | ILMN_1780663 | - | not significant | 3.7 | not significant | 7.8 | not significant | - | not significant |
| ***MGC16025*** | 2 | ILMN_1757561 | 0.7 | not significant | - | not significant | 1.1 | not significant | - | not significant |
| ***PER2*** | 2 | ILMN_1738095 | 93.8 | 2.64E-03 | 129.1 | < 10E-03 | 112.4 | < 10E-03 | 50.3 | 3.43E-02 |
| ***SCLY*** | 2 | ILMN_1722742 | 1.0 | not significant | 7.1 | not significant | - | not significant | 35.5 | not significant |
| ***TRAF3IP1*** | 2 | ILMN_2067032 | 22.5 | 2.24E-02 | 6.7 | not significant | 17.7 | 1.19E-02 | 4.3 | not significant |
| ***UBE2F*** | 2 | ILMN_2164242 | 257.6 | 1.32E-03 | 325.6 | < 10E-03 | 256.3 | < 10E-03 | 584.5 | < 10E-03 |
| ***AUTS2*** | 7 | ILMN_1749081 | 57.3 | 2.64E-03 | 331.4 | < 10E-03 | 270.0 | < 10E-03 | 9.5 | not significant |
| ***FERD3L*** | 7 | ILMN_1721475 | - | not significant | - | not significant | - | not significant | 5.4 | not significant |
| ***HDAC9*** | 7 | ILMN_2408885 | 33.7 | 6.59E-03 | 8.8 | not significant | - | not significant | 138.4 | < 10E-03 |
| ***PMS2L4*** | 7 | no probe | no probe | no probe | no probe | no probe | no probe | no probe | no probe | no probe |
| ***STAG3L4*** | 7 | ILMN_1669851 | 3.4 | not significant | 16.7 | 7.91E-03 | 6.8 | not significant | 4.5 | not significant |
| ***TWIST1*** | 7 | ILMN_1672908 | 43.7 | 2.64E-03 | 129.1 | < 10E-03 | 107.3 | < 10E-03 | 15.9 | not significant |
| ***TYW1*** | 7 | ILMN_1736135 | 57.4 | 2.64E-03 | 22.2 | 2.64E-03 | 36.2 | < 10E-03 | 45.1 | 4.08E-02 |
| ***WBSCR17*** | 7 | ILMN_1701557 | 1.1 | not significant | 0.1 | not significant | - | not significant | - | not significant |
| ***C17orf69*** | 17 | ILMN_1743621 | 14.6 | not significant | 12.3 | 3.29E-02 | 17.7 | 1.19E-02 | - | not significant |
| ***CRHR1*** | 17 | no probe | no probe | no probe | no probe | no probe | no probe | no probe | no probe | no probe |
| ***IMP5*** | 17 | ILMN_1744094 | 4.2 | not significant | 15.7 | 1.05E-02 | 11.9 | 3.69E-02 | 83.9 | 6.59E-03 |
| ***KIAA1267*** | 17 | ILMN_2200636 | 359.7 | < 10E-03 | 336.7 | < 10E-03 | 388.0 | < 10E-03 | 427.8 | < 10E-03 |
| ***LOC100128977*** | 17 | no probe | no probe | no probe | no probe | no probe | no probe | no probe | no probe | no probe |
| ***LOC100130148*** | 17 | no probe | no probe | no probe | no probe | no probe | no probe | no probe | no probe | no probe |
| ***LOC644172*** | 17 | ILMN_1772603 | 8.6 | not significant | 12.2 | 3.29E-02 | 3.1 | not significant | 14.3 | not significant |
| ***LRRC37A4*** | 17 | ILMN_2393693 | 3.7 | not significant | 18.9 | 6.59E-03 | 13.1 | 2.77E-02 | 20.0 | not significant |
| ***MAPT*** | 17 | ILMN_2298727 | 29.8 | 9.22E-03 | - | not significant | 2.0 | not significant | 33.4 | not significant |
| ***MGC57346*** | 17 | ILMN_1784428 | 90.4 | 2.64E-03 | 22.4 | 2.64E-03 | 44.1 | < 10E-03 | 48.0 | 3.95E-02 |
| ***PLEKHM1*** | 17 | ILMN_1709549 | 21.9 | 2.37E-02 | 11.8 | 3.56E-02 | 34.5 | < 10E-03 | 121.8 | 2.64E-03 |
| ***STH*** | 17 | ILMN_1665311 | 9.8 | not significant | 4.4 | not significant | 12.9 | 3.03E-02 | 0.9 | not significant |
| ***KIAA1632*** | 18 | ILMN_1762608 | - | not significant | 11.2 | 4.08E-02 | 17.9 | 1.19E-02 | - | not significant |
| ***PSTPIP2*** | 18 | ILMN_1713058 | 53.7 | 2.64E-03 | 50.6 | < 10E-03 | 46.5 | < 10E-03 | 128.7 | 1.32E-03 |
| ***SETBP1*** | 18 | ILMN_1720513 | 103.4 | 2.64E-03 | 83.2 | < 10E-03 | 124.1 | < 10E-03 | - | not significant |
| ***SIGLEC15*** | 18 | ILMN_1797293 | - | not significant | - | not significant | - | not significant | - | not significant |
| ***SLC14A1*** | 18 | ILMN_1805561 | - | not significant | 27.7 | < 10E-03 | 12.6 | 3.03E-02 | 62.2 | 2.11E-02 |
| ***SLC14A2*** | 18 | ILMN_1664320 | - | not significant | 6.9 | not significant | - | not significant | - | not significant |
| ***C20orf56*** | 20 | no probe | no probe | no probe | no probe | no probe | no probe | no probe | no probe | no probe |
| ***FOXA2*** | 20 | ILMN_1668052 | - | not significant | - | not significant | - | not significant | 6.0 | not significant |
| ***LOC284788*** | 20 | ILMN_1823704 | - | not significant | - | not significant | - | not significant | - | not significant |
| ***NKX2-2*** | 20 | ILMN_1692280 | - | not significant | - | not significant | - | not significant | 4.4 | not significant |
| ***NKX2-4*** | 20 | no probe | no probe | no probe | no probe | no probe | no probe | no probe | no probe | no probe |
| ***PAX1*** | 20 | ILMN_1682096 | 1.7 | not significant | - | not significant | - | not significant | 8.7 | not significant |
| ***PLK1S1*** | 20 | ILMN_1779536 | 20.7 | 2.50E-02 | 13.7 | 2.24E-02 | 20.8 | 9.22E-03 | 11.2 | not significant |
| ***SSTR4*** | 20 | ILMN_1684200 | 5.4 | not significant | - | not significant | - | not significant | 0.5 | not significant |
| ***THBD*** | 20 | ILMN_1759787 | 38.7 | 3.95E-03 | 18.5 | 6.59E-03 | 15.4 | 1.98E-02 | 31.9 | not significant |
| ***XRN2*** | 20 | ILMN_1727617 | 40.7 | 3.95E-03 | 73.2 | < 10E-03 | 33.3 | < 10E-03 | 175.7 | < 10E-03 |

Abbreviations: Chr., chromosome; AVG, average.
