# Supplementary material for: Six Novel Susceptibility Loci for Early-Onset Androgenetic Alopecia and Their Unexpected Association with Common Diseases
Source: PLoS Genet. 2012 May 31;8(5):e1002746. doi: 10.1371/journal.pgen.1002746 (PMC3364959; doi:10.1371/journal.pgen.1002746)
Supplement: Table S4 — Summary result for the top SNPs from the genome-wide significant loci using random effect models. (DOC) [file pgen.1002746.s007.doc]

**Table S4 Summary result for the top SNPs from the genome-wide significant loci using random effect models.**

| Genetic  Variant | Chr. | Position^a^ | EA^b^/  NEA | EAF | OR (95% CI) | p value | Gene^c^ | Number of  GW Significant SNPs | Q p value | *I*^2^ |
| --- | --- | --- | --- | --- | --- | --- | --- | --- | --- | --- |
| rs12565727 | 1 | 10955669 | A/G | 0.789 | 1.33 (1.22-1.45) | 9.07 x 10^-11^ | *TARDBP* | 8 | 0.43 | < 0.01 |
| rs11683401 | 2 | 239362355 | T/C | 0.447 | 1.26 (1.18-1.35) | 6.02 x 10^-11^ | *HDAC4* | 9 | 0.60 | < 0.01 |
| rs2073963 | 7 | 18844399 | G/T | 0.530 | 1.29 (1.20-1.38) | 1.27 x 10^-12^ | *HDAC9* | 4 | 0.42 | < 0.01 |
| rs6945541 | 7 | 68249896 | C/T | 0.539 | 1.27 (1.18-1.38) | 1.71 x 10^-9^ | *AUTS2* | 6 | 0.97 | < 0.01 |
| rs17650991 | 17 | 41400344 | A/C | 0.799 | 1.29 (1.19-1.41) | 6.85 x 10^-9^ | 17q21.31 | 88 | 0.45 | < 0.01 |
| rs6047844 | 20 | 21985575 | T/C | 0.460 | 1.60 (1.49-1.72) | 1.71 x 10^-39^ | *PAX1, FOXA2* | 193 | 0.58 | < 0.01 |
| rs1511061 | X | 66232849 | A/G | 0.822 | 2.40 (2.21-2.62) | 6.82 x 10^-90^ | *AR* | 177 | 0.71 | <0.01 |

Abbreviations: Chr., chromosome; EA, effect allele; NEA, non-effect allele; EAF, effect allele frequency; OR, odds ratio; CI, confidence interval.

^a^-Chromosome position located in reference sequence of Genome Build 36.3. ^b^-AGA risk increasing allele on forward strand. ^c^-Gene harboring the SNP or nearest to the SNP.
