# Supplementary material for: Six Novel Susceptibility Loci for Early-Onset Androgenetic Alopecia and Their Unexpected Association with Common Diseases
Source: PLoS Genet. 2012 May 31;8(5):e1002746. doi: 10.1371/journal.pgen.1002746 (PMC3364959; doi:10.1371/journal.pgen.1002746)
Supplement: Table S5 — Interaction results between top SNPs of AR locus and other AGA loci. (DOC) [file pgen.1002746.s008.doc]

**Table S5** Interaction results between top SNPs of *AR* locus and other AGA loci

| Chr. | SNP | Interaction  OR (95% CI) | Interaction  p value | *I^2^* |
| --- | --- | --- | --- | --- |
| 1 | rs12565727 | 0.99 (0.94-1.05) | 0.83 | < 0.01 |
| 2 | rs9287638 | 1.03 (0.99-1.07) | 0.10 | < 0.01 |
| 7 | rs2073963 | 1.01 (0.98-1.05) | 0.42 | < 0.01 |
| 7 | rs6945541^a^ | 1.04 (1.00-1.09) | 0.07 | 0.57 |
| 17 | rs12373124 | 1.00 (0.92-1.09) | 0.97 | < 0.01 |
| 18 | rs10502861 | 1.01 (0.97-1.04) | 0.75 | 0.38 |
| 20 | rs6047844 | 1.02 (0.99-1.05) | 0.31 | < 0.01 |

Abbreviations: Chr., chromosome; OR, odds ratio; CI, confidence interval.

^a^-*I^2^* is greater than 50%, meta-analysis was also performed under random effect model with OR (95% CI) = 1.07 (0.98-1.16), *P* value = 0.14.
