# Supplementary material for: Six Novel Susceptibility Loci for Early-Onset Androgenetic Alopecia and Their Unexpected Association with Common Diseases
Source: PLoS Genet. 2012 May 31;8(5):e1002746. doi: 10.1371/journal.pgen.1002746 (PMC3364959; doi:10.1371/journal.pgen.1002746)
Supplement: Table S6 — Association test results for AGA, at Parkinson's disease loci. (DOC) [file pgen.1002746.s009.doc]

**Table S6** Association test results for AGA, at Parkinson’s disease loci

| **SNP** | **chr** | **position** | **NEA/**  **EA** | **Gt.**  **rate** | **EAF** | **HWE**  **p value** | **p value** | **Odds**  **Ratio** | **95% CI** | **Gene** |
| --- | --- | --- | --- | --- | --- | --- | --- | --- | --- | --- |
| rs34637584 | 12 | 39020469 | A/G | 1.000 | 0.998 | 0.012 | 0.56 | 0.769 | 0.314,1.886 | *LRRK2* |
| rs356219 | 4 | 90856624 | A/G | 0.974 | 0.370 | 0.75 | 0.86 | 0.993 | 0.919,1.073 | *GPRIN3-SNCA* |
| i4000415 | 1 | 153472258 | C/T | 0.991 | 0.995 | 0.59 | 0.34 | 0.766 | 0.441,1.331 | *GBA* |
| **rs12185268** | **17** | **41279463** | **A/G** | **0.988** | **0.217** | **0.32** | **9.3e-08** | **0.782** | **0.714,0.856** | ***IMP5*** |
| rs10513789 | 3 | 184242767 | G/T | 1.000 | 0.796 | 0.78 | 0.39 | 1.041 | 0.950,1.140 | *MCCC1* |
| rs6812193 | 4 | 77418010 | C/T | 1.000 | 0.368 | 0.93 | 0.27 | 0.958 | 0.887,1.034 | *FAM47D* |
| rs823156 | 1 | 204031263 | A/G | 0.999 | 0.180 | 0.32 | 0.44 | 0.963 | 0.876,1.059 | *SLC41A1* |
| rs7599054 | 2 | 135257016 | A/G | 1.000 | 0.462 | 0.0032 | 0.27 | 0.958 | 0.887,1.034 | *TMEM163-ACMSD* |
| rs6599389 | 4 | 929113 | A/G | 1.000 | 0.925 | 0.56 | 0.32 | 0.932 | 0.810,1.072 | *TMEM175* |
| rs11868035 | 17 | 17655826 | A/G | 0.999 | 0.686 | 0.051 | 0.23 | 1.049 | 0.970,1.134 | *RAI1-SREBF1* |
| rs10886515 | 10 | 121333579 | C/T | 0.999 | 0.712 | 0.60 | 0.50 | 0.972 | 0.897,1.054 | *TIAL1* |
| rs6475667 | 9 | 22598036 | A/G | 1.000 | 0.135 | 0.34 | 0.72 | 0.980 | 0.881,1.091 | *DMRTA1* |
| rs660895 | 6 | 32685358 | A/G | 1.000 | 0.191 | 0.052 | 0.74 | 1.016 | 0.924,1.117 | *HLA-DRB5-HLA-DQA1* |
| rs2823357 | 21 | 15836776 | A/G | 0.999 | 0.620 | 0.19 | 0.44 | 0.970 | 0.899,1.047 | *NRIP1-USP25* |
| rs11065598 | 12 | 120418046 | C/T | 0.997 | 0.095 | 0.73 | 0.61 | 0.968 | 0.853,1.097 | *FBXL10* |
| rs9379968 | 6 | 27352569 | A/G | 1.000 | 0.526 | 0.94 | 0.56 | 0.978 | 0.909,1.053 | *PRSS16-FKSG83* |
| rs9917256 | 2 | 168851281 | A/G | 0.999 | 0.875 | 0.70 | 0.40 | 1.049 | 0.939,1.172 | *STK39-LASS6* |
| rs10999435 | 10 | 72045807 | A/G | 1.000 | 0.927 | 0.23 | 0.57 | 0.960 | 0.833,1.105 | *PRF1-ADAMTS14* |
| rs4130047 | 18 | 38932233 | C/T | 1.000 | 0.682 | 0.89 | 0.60 | 0.979 | 0.905,1.060 | *RIT2* |
| rs35883 | 3 | 29572795 | A/G | 1.000 | 0.546 | 0.95 | 0.52 | 0.976 | 0.907,1.051 | *RBMS3* |
| rs943914 | 14 | 54509818 | A/C | 0.997 | 0.705 | 0.47 | 0.059 | 1.080 | 0.997,1.171 | *WDHD1* |
| rs7778020 | 7 | 48427057 | A/G | 0.999 | 0.905 | 0.94 | 0.41 | 0.949 | 0.837,1.076 | *ABCA13* |
| rs535586 | 6 | 31968316 | C/T | 0.703 | 0.309 | 0.018 | 0.16 | 1.072 | 0.974,1.179 | *EHMT2* |
| rs17763599 | 19 | 2320415 | G/T | 1.000 | 0.279 | 0.38 | 0.31 | 1.043 | 0.962,1.132 | *SPPL2B-TMPRSS9* |
| rs2414739 | 15 | 59781426 | A/G | 1.000 | 0.269 | 0.86 | 0.59 | 0.977 | 0.899,1.062 | *VPS13C* |
| rs624032 | 10 | 28998382 | C/T | 0.998 | 0.355 | 0.85 | 0.15 | 1.058 | 0.980,1.143 | *WAC-BAMBI* |
| rs182549 | 2 | 136333224 | C/T | 1.000 | 0.578 | 2.4e-29 | 0.89 | 1.006 | 0.926,1.092 | *MCM6* |
| rs6464536 | 7 | 142399594 | C/T | 0.999 | 0.085 | 0.92 | 0.84 | 1.014 | 0.888,1.157 | *KEL-OR9A2* |
| rs7705606 | 5 | 39243174 | G/T | 0.357 | 0.999 | 0.93 | 0.49 | 0.554 | 0.099,3.088 | *FYB* |
| rs17126237 | 8 | 18025369 | C/T | 0.998 | 0.118 | 0.57 | 0.64 | 1.028 | 0.916,1.154 | *ASAH1-NAT1* |

Abbreviation: chr, chromosome; NEA, non-effect allele; EA, effect allele; Gt. rate, genotyping rate; EAF, effect allele frequency.

Note: significant SNP is in bold
