# Supplementary material for: Six Novel Susceptibility Loci for Early-Onset Androgenetic Alopecia and Their Unexpected Association with Common Diseases
Source: PLoS Genet. 2012 May 31;8(5):e1002746. doi: 10.1371/journal.pgen.1002746 (PMC3364959; doi:10.1371/journal.pgen.1002746)
Supplement: Table S7 — Association test results for Parkinson's disease, at AGA loci. (DOC) [file pgen.1002746.s010.doc]

**Table S7 Association test results for Parkinson’s disease, at AGA loci**

| **SNP** | **chr** | **position** | **NEA/**  **EA** | **Gt.**  **rate** | **EAF** | **HWE**  **p value** | **p value** | **Odds**  **Ratio** | **95% CI** | **Gene** |
| --- | --- | --- | --- | --- | --- | --- | --- | --- | --- | --- |
| rs5918696 | X | 66242280 | C/T | 0.999 | 0.148 | 0.15 | 0.54 | 1.020 | 0.958,1.085 | *EDA2R-AR* |
| rs6113491 | 20 | 22005415 | A/C | 1.000 | 0.489 | 0.037 | 0.82 | 1.007 | 0.952,1.064 | *PAX1-FOXA2* |
| rs13245206 | 7 | 18857784 | A/G | 1.000 | 0.608 | 0.047 | 0.19 | 1.039 | 0.981,1.100 | *HDAC9* |
| rs9287638 | 2 | 239359379 | A/C | 1.000 | 0.676 | 0.12 | 0.51 | 0.980 | 0.924,1.040 | *ASB1-HDAC4* |
| rs2003046 | 1 | 10955414 | A/C | 1.000 | 0.772 | 0.13 | 0.38 | 1.030 | 0.963,1.102 | *C1orf127-TARDBP* |
| rs6950087 | 7 | 68233508 | C/T | 0.999 | 0.538 | 0.046 | 0.33 | 0.973 | 0.921,1.029 | *AUTS2* |
| rs7349332 | 2 | 219464627 | C/T | 1.000 | 0.136 | 0.22 | 0.79 | 1.011 | 0.932,1.097 | *WNT10A* |
| **rs2668692** | **17** | **41648797** | **A/G** | **0.992** | **0.784** | **0.14** | **2.8e-12** | **1.283** | **1.195,1.378** | ***KIAA1267-LRRC37A*** |
| rs9668810 | 12 | 26317687 | C/T | 1.000 | 0.265 | 0.0010 | 0.62 | 1.016 | 0.954,1.082 | *SSPN-ITPR2* |
| rs11079281 | 17 | 52586786 | C/T | 1.000 | 0.539 | 0.45 | 0.72 | 1.010 | 0.955,1.069 | *AKAP1-MSI2* |
| rs8085664 | 18 | 41068154 | A/C | 0.999 | 0.726 | 0.66 | 0.65 | 1.015 | 0.953,1.081 | *SETBP1-SLC14A2* |
| rs10850917 | 12 | 116801217 | C/T | 1.000 | 0.240 | 0.89 | 0.58 | 1.019 | 0.954,1.088 | *KSR2* |
| rs10935880 | 3 | 153137969 | A/G | 1.000 | 0.713 | 0.86 | 0.58 | 1.018 | 0.957,1.082 | *SUCNR1-MBNL1* |
| rs7707918 | 5 | 158202803 | A/C | 1.000 | 0.403 | 0.029 | 0.59 | 1.016 | 0.960,1.075 | *EBF1* |
| rs2700482 | 12 | 49369532 | C/T | 1.000 | 0.349 | 0.77 | 0.32 | 1.030 | 0.972,1.092 | *DIP2B* |
| rs12194169 | 6 | 9601288 | G/T | 1.000 | 0.051 | 0.034 | 0.66 | 1.029 | 0.906,1.168 | *OFCC1* |
| rs1907354 | 10 | 77882399 | A/G | 0.998 | 0.679 | 0.26 | 0.31 | 0.969 | 0.913,1.029 | *C10orf11* |
| rs13130633 | 4 | 8345096 | C/T | 0.679 | 0.226 | 0.27 | 0.051 | 1.073 | 1.000,1.151 | *HTRA3* |
| rs803307 | 1 | 25331538 | A/G | 1.000 | 0.336 | 0.25 | 0.37 | 0.973 | 0.917,1.033 | *RUNX3-SYF2* |
| rs10812596 | 9 | 27435104 | A/G | 0.679 | 0.301 | 0.67 | 0.78 | 0.991 | 0.929,1.057 | *MOBKL2B* |
| rs4940205 | 18 | 43291571 | A/G | 0.999 | 0.281 | 0.19 | 0.65 | 1.015 | 0.954,1.080 | *CORL2-SMAD2* |
| rs12026324 | 1 | 119248233 | C/T | 1.000 | 0.709 | 0.26 | 0.79 | 0.992 | 0.932,1.054 | *TBX15* |
| rs8053509 | 16 | 8769772 | G/T | 0.999 | 0.865 | 0.12 | 0.98 | 0.999 | 0.921,1.084 | *ABAT* |
| rs2861754 | 2 | 67931211 | A/G | 1.000 | 0.654 | 0.74 | 0.37 | 1.027 | 0.968,1.090 | *ETAA1-C1D* |
| rs5762174 | 22 | 26195344 | A/C | 0.998 | 0.390 | 0.15 | 0.49 | 0.980 | 0.925,1.038 | *CRYBA4-MN1* |
| rs4733710 | 8 | 130612833 | A/C | 1.000 | 0.747 | 0.96 | 0.14 | 1.050 | 0.985,1.120 | *GSDMC* |

Abbreviation: chr, chromosome; NEA, non-effect allele; EA, effect allele; Gt. rate, genotyping rate; EAF, effect allele frequency.
