# Supplementary material for: Six Novel Susceptibility Loci for Early-Onset Androgenetic Alopecia and Their Unexpected Association with Common Diseases
Source: PLoS Genet. 2012 May 31;8(5):e1002746. doi: 10.1371/journal.pgen.1002746 (PMC3364959; doi:10.1371/journal.pgen.1002746)
Supplement: Text S1 — Description of participant studies and phenotype definition. Methods of tissue expression analysis are also described. (DOC) [file pgen.1002746.s011.doc]

**Six Novel Susceptibility Loci for Early-Onset Androgenetic Alopecia and Their Association with Unexpected Common Diseases**

Rui Li PhD,[^1^](#_ENREF_1)* Felix F Brockschmidt PhD,^2-3^* Amy K Kiefer PhD,[^4^](#_ENREF_4)* Hreinn Stefansson PhD,[^5^](#_ENREF_5)* Dale R Nyholt PhD,[^6^](#_ENREF_6)* Kijoung Song PhD,[^7^](#_ENREF_7)* Sita H. Vermeulen PhD,^8-9^* Stavroula Kanoni PhD,[^10^](#_ENREF_10)* Daniel Glass PhD,[^11^](#_ENREF_11) Sarah E Medland PhD,[^6^](#_ENREF_6) Maria Dimitriou PhD,[^12^](#_ENREF_12) Dawn Waterworth PhD,[^7^](#_ENREF_7) Joyce Y Tung PhD,[^4^](#_ENREF_4) Frank Geller MSc,[^13^](#_ENREF_13) Stefanie Heilmann PhD,^2-3^ Axel M Hillmer PhD,^14^ Veronique Bataille MD,[^11^](#_ENREF_11) Sibylle Eigelshoven MD,[^15^](#_ENREF_15) Sandra Hanneken MD,[^15^](#_ENREF_15) Susanne Moebus PhD,[^16^](#_ENREF_16) Christine Herold PhD,[^17^](#_ENREF_17) Martin den Heijer MD,[^18^](#_ENREF_18) Grant W Montgomery PhD,[^6^](#_ENREF_6) Panos Deloukas PhD,[^10^](#_ENREF_10) Nicholas Eriksson PhD,[^4^](#_ENREF_4) Andrew C Heath PhD,[^19^](#_ENREF_19) Tim Becker PhD,^17, 20^ Patrick Sulem PhD,[^5^](#_ENREF_5) Massimo Mangino PhD,[^11^](#_ENREF_11) Peter Vollenweider MD,[^21^](#_ENREF_21) Tim D Spector MD,[^11^](#_ENREF_11) George Dedoussis PhD,[^12^](#_ENREF_12)§ Nicholas G Martin PhD,[^6^](#_ENREF_6)§ Lambertus A. Kiemeney PhD,^8, 22-23^§ Vincent Mooser MD,[^7^](#_ENREF_7)§ Kari Stefansson MD,[^5^](#_ENREF_5)§ David A Hinds PhD,[^4^](#_ENREF_4)§ Markus M Nöthen MD,^2-3, 17^§, and J Brent Richards MD^1, 11^§

* and § denote equal contribution

1. Departments of Medicine, Human Genetics, Epidemiology and Biostatistics, Lady Davis Institute, Jewish General Hospital, McGill University, Montreal, Quebec, Canada.

2. Institute of Human Genetics, University of Bonn, D-53127 Bonn, Germany.

3. Department of Genomics, Life & Brain Center, University of Bonn, D-53127 Bonn, Germany.

4. 23andMe, Mountain View, CA.

5. deCODE genetics, Sturlugata 8 IS-101, Reykjavík, Iceland.

6. Queensland Institute of Medical Research, Brisbane 4029, Australia.

7. Genetics Division, GlaxoSmithKline, King of Prussia, Pennsylvania 19406, USA.

8. Department of Epidemiology, Biostatistics and HTA, Radboud University Nijmegen Medical Centre, PO Box 9101, 6500 HB, Nijmegen, the Netherlands

9. Department of Genetics, Radboud University Nijmegen Medical Centre, PO Box 9101, 6500 HB, Nijmegen, the Netherlands

10. Genetics of complex traits in humans, Wellcome Trust Sanger Institute, Wellcome Trust Genome Campus, Hinxton, CB10 1SA, UK.

11. Twin Research and Genetic Epidemiology, King’s College London, London, United Kingdom.

12. Department of Dietetics-Nutrition, Harokopio University, Athens, Greece.

13. Department of Epidemiology Research, Statens Serum Institute, Artillerivej 5, 2300 Copenhagen S, Denmark.

14. Genome Technology & Biology, Genome Institute of Singapore, Singapore 138672, Singapore.

15. Department of Dermatology, University of Düsseldorf, D-40225 Düsseldorf, Germany.

16. Institute for Medical Informatics, Biometry and Epidemiology, University Clinic Essen, University Duisburg-Essen, 45122 Essen, Germany.

17. German Center for Neurodegenerative Diseases (DZNE), D-53127 Bonn, Germany.

18. Department of Endocrinology, Radboud University Nijmegen Medical Centre, PO Box 9101, 6500 HB, Nijmegen, the Netherlands

19. Washington University Medical School, St Louis , USA.

20. Institute for Medical Biometry, Informatics and Epidemiology, University of Bonn, Sigmund-Freud-Str. 25, D-53127

21. Department of Internal Medicine, CHUV University Hospital, Lausanne 1011, Switzerland.

22. Department of Urology, Radboud University Nijmegen Medical Centre, PO Box 9101, 6500 HB, Nijmegen, the Netherlands

23. IKNL, Location Nijmegen, PO Box 1281, 6501 BG, Nijmegen the Netherlands

Correspondence to: J Brent Richards. Mailing address: Pavilion H, Jewish General Hospital, 3755 Cote Ste Catherine Ouest, Montreal, Quebec H3T 1E2, Canada. Email address: [brent.richards@mcgill.ca](mailto:brent.richards@mcgill.ca). Tel: 1-514-340-8222 Ext: 4362.

**Table of Contents**

**1 Description of participant studies and phenotype definition**

**2 Tissue expression analysis**

**1 Description of participant studies and phenotype definition**

**Bonn**: All cases were recruited by dermatologists at the Department of Dermatology, University of Düsseldorf, Germany. Males who were <30 years of age having AGA of grades IV-VII or <40 years having AGA of grades V-VII[1]. Population-based controls were recruited at the University of Essen for the Heinz Nixdorf Recall cohort (Risk Factors, Evaluation of Coronary Calcium and Lifestyle). All the cases and controls were of German descent and their baldness status were assessed by dermatologist and documented by photographs. The current sample size consists of 582 cases and 347 controls.

**CoLaus**: Members of this cohort were randomly selected from permanent residents of Lausanne, Switzerland between 2003 and 2006[2,3]. Only individuals with 4 European grandparents were eligible for inclusion in the GWA study. Men were considered to have androgenic alopecia if they experienced Hamilton grade V-VII alopecia between the ages of 35 and 65, while controls were men displaying no hair loss (Hamilton grade I) between ages 45 and 55 or grade II between the ages of 56 and 75. Age of onset was assessed by questionnaire. Participants were recruited and assessed by nurses who had been specifically trained to assess for androgenic alopecia. The current study sample consists of 578 cases and 547 controls.

**TwinsUK**: The TwinsUK cohort is a population-based sample of Britons, unselected for any disease or trait, which is representative of the UK singleton population[4]. A dermatologist using standardized photographs assessed all subjects. Cases were defined as Hamilton grade V-VII and controls were those without androgenic alopecia (Hamilton grade I). The current study included 162 cases and 210 controls[5].

**Nijmegen Biomedical Study.** The details of this study were reported previously[6]. Briefly, this is a population-based survey conducted by the Department of Epidemiology and Biostatistics and the Department of Clinical Chemistry of the Radboud University Nijmegen Medical Center (RUNMC), in which 9,371 individuals participated from a total of 22,500 age and sex stratified, randomly selected inhabitants of Nijmegen. Individuals in the Nijmegen Biomedical Study were invited to participate in a study on gene-environment interactions in multifactorial diseases, such as cancer. All participants are of self-reported European descent. In a second phase of this project, participants completed an additional questionnaire and matched their hair pattern at 20 years, 40 years and present age to a Hamilton grading schema[7]. Affected individuals were those reporting Hamilton grade IV-VII by age 40 and controls were those with Hamilton grade I at age 50 years or older, or Hamilton grade II-III at age 60 years or older. A total of 73 cases and 132 controls were included in the current analyses.

**23andMe**: Participants in this study are unrelated male individuals of predominantly European ancestry, selected from users of the 23andMe Personal Genome Service[8]. Status of male pattern baldness was measured by self assessment through a web based survey. Individuals with Hamilton grade III or higher, age of onset < 40 were defined as cases, and individuals with Hamilton grade I and age >= 30, or Hamilton grade II and age >= 50 were considered as controls. In the current study 2,167 cases and 1,753 controls were included.

**Iceland**: The hair loss of study subjects and the age of onset were identified from questionnaire data. Individuals with considerable hair loss before the age of 50 years were defined as cases. Individuals older than 50 years who reported no hair loss were defined as controls. The current study includes 191 cases and 198 controls.

**Australian population based twin study**: Measures of hair loss were obtained in the course of an extensive semi-structured telephone interview with respondent booklet, designed to assess physical, psychological and social manifestations of alcoholism and related disorders, conducted with 6265 twins born 1964-71 from the volunteer based Australian Twin Registry. All males (45% of the sample) were asked to rate their degree of hair loss, if any, using the Hamilton-Norwood Baldness scale, which was printed in the respondent booklet). This data collection scheme was validated in a study by Ellis et al.[9]. Individuals with hair loss Hamilton-Norwood type III or greater were classified as cases and population based individuals were recruited as controls. The current analyses consist of 138 unrelated cases and 5728 unrelated controls.

**THISEAS**: The THISEAS study is a case- control study for coronary artery disease. As part of the study, hair status was assessed in male individuals by trained scientists. The pattern of AGA was determined using the Hamilton and Norwood classification. The controls were those with no hair loss or those with Hamilton grade II at the age of 50 years or older. Participants with Hamilton grade III or higher, age of onset < 50 years were defined as cases. 297 controls and 219 cases were included in the analyses of the current study.

**2 Tissue expression analysis**

Total RNA from human hair follicles, skin from temple, scalp and whole blood was extracted (RNeasy Micro Kit, Qiagen, Hilden, Germany). Quality and quantity of the RNA was analyzed on a NanoDrop ND-1000 spectrophotometer (Peqlab Biotechnologie, Erlangen, Germany). For quality reasons, each RNA sample was additionally checked for degradation via gel electrophoresis in a BioAnalyzer 2100 (Agilent Technologies, Waldbronn, Germany) with RNA 6000 nano lab chips following manufacturer’s instructions. Array-based gene expression analysis of individual tissue samples was performed on Illumina’s human HT-12v3 Expression BeadChips using standard protocols (Illumina, San Diego, USA). In brief, 50ng of total RNA was reverse transcribed into cRNA and biotin-UTP labelled using the Illumina TotalPrep 96-RNA Amplification Kit (Ambion/Applied Biosystems, Darmstadt, Germany). Labelled cRNA was hybridized on Illumina HT-12v3 Expression BeadChips.

The obtained fluorescence expression data were background subtracted and quantile normalized using GenomeStudio software (Illumina, San Diego, USA). Supplementary Table3 lists all genes shown in Figure 1 that neighbour the SNP achieving the most significant p-value at the respective identified risk loci for AGA. The average signal (AVG-signal) of identical probes and the detection p-values (which is significant, if a gene is reliably expressed) are shown for each transcript tested. If the expression of a respective gene was detected by more than one Illumina probe, the probe with the best expression values based on all samples tested is presented. The same applies, if several probes detected different transcript variants of a respective gene. Non-present fluorescence intensity after background subtraction was marked as “-“ and defined as not expressed in the corresponding tissue.

References

1. Hillmer AM, Brockschmidt FF, Hanneken S, Eigelshoven S, Steffens M, et al. (2008) Susceptibility variants for male-pattern baldness on chromosome 20p11. Nat Genet 40: 1279-1281.

2. Firmann M, Mayor V, Vidal PM, Bochud M, Pecoud A, et al. (2008) The CoLaus study: a population-based study to investigate the epidemiology and genetic determinants of cardiovascular risk factors and metabolic syndrome. BMC Cardiovasc Disord 8: 6.

3. Sandhu MS, Waterworth DM, Debenham SL, Wheeler E, Papadakis K, et al. (2008) LDL-cholesterol concentrations: a genome-wide association study. Lancet 371: 483-491.

4. Andrew T, Hart DJ, Snieder H, de Lange M, Spector TD, et al. (2001) Are twins and singletons comparable? A study of disease-related and lifestyle characteristics in adult women. Twin Res 4: 464-477.

5. Richards JB, Yuan X, Geller F, Waterworth D, Bataille V, et al. (2008) Male-pattern baldness susceptibility locus at 20p11. Nat Genet 40: 1282-1284.

6. Wetzels JF, Kiemeney LA, Swinkels DW, Willems HL, den Heijer M (2007) Age- and gender-specific reference values of estimated GFR in Caucasians: the Nijmegen Biomedical Study. Kidney Int 72: 632-637.

7. Gudmundsson J, Sulem P, Steinthorsdottir V, Bergthorsson JT, Thorleifsson G, et al. (2007) Two variants on chromosome 17 confer prostate cancer risk, and the one in TCF2 protects against type 2 diabetes. Nat Genet 39: 977-983.

8. Eriksson N, Macpherson JM, Tung JY, Hon LS, Naughton B, et al. (2010) Web-based, participant-driven studies yield novel genetic associations for common traits. PLoS Genet 6: e1000993.

9. Ellis JA, Stebbing M, Harrap SB (1998) Genetic analysis of male pattern baldness and the 5alpha-reductase genes. J Invest Dermatol 110: 849-853.
